# Bayesian variable selection with graphical structure learning: Applications in integrative genomics

**DOI:** 10.1371/journal.pone.0195070

**Published:** 2018-07-30

**Authors:** Suprateek Kundu, Yichen Cheng, Minsuk Shin, Ganiraju Manyam, Bani K. Mallick, Veerabhadran Baladandayuthapani

**Affiliations:** 1 Department of Biostatistics & Bioinformatics, Emory University, 1518 Clifton Road. Atlanta, Georgia, 30322, United States of America; 2 Robinson College of Business, Georgia State University, 35 Broad St. NW, Atlanta, Georgia, 30303, United States of America; 3 Department of Statistics, Texas A&M, 155 Ireland St., College Station, Texas, 77843, United States of America; 4 Department of Biostatistics, M.D. Anderson Cancer Research Center, Houston, Texas, 77030, United States of America; University of Miami, UNITED STATES

## Abstract

Significant advances in biotechnology have allowed for simultaneous measurement of molecular data across multiple genomic, epigenomic and transcriptomic levels from a single tumor/patient sample. This has motivated systematic data-driven approaches to integrate multi-dimensional structured datasets, since cancer development and progression is driven by numerous co-ordinated molecular alterations and the interactions between them. We propose a novel multi-scale Bayesian approach that combines integrative graphical structure learning from multiple sources of data with a variable selection framework—to determine the key genomic drivers of cancer progression. The integrative structure learning is first accomplished through novel joint graphical models for heterogeneous (mixed scale) data, allowing for flexible and interpretable incorporation of prior existing knowledge. This subsequently informs a variable selection step to identify groups of co-ordinated molecular features within and across platforms associated with clinical outcomes of cancer progression, while according appropriate adjustments for multicollinearity and multiplicities. We evaluate our methods through rigorous simulations to establish superiority over existing methods that do not take the network and/or prior information into account. Our methods are motivated by and applied to a glioblastoma multiforme (GBM) dataset from The Cancer Genome Atlas to predict patient survival times integrating gene expression, copy number and methylation data. We find a high concordance between our selected prognostic gene network modules with known associations with GBM. In addition, our model discovers several novel cross-platform network interactions (both cis and trans acting) between gene expression, copy number variation associated gene dosing and epigenetic regulation through promoter methylation, some with known implications in the etiology of GBM. Our framework provides a useful tool for biomedical researchers, since clinical prediction using multi-platform genomic information is an important step towards personalized treatment of many cancers.

## Introduction

The last decade has seen a proliferation of multi-platform genomic data, aided partly by the rapid evolution and declining costs of modern technologies, producing high-throughput multi-dimensional data. It is now technologically and economically feasible to collect diverse data on matched patient/tumor samples at a detailed molecular resolution across multiple modalities such as genomics (DNA copy number), epigenomics (methylation), transcriptomics (mRNA/gene expression) and proteomics. Such large scale coordinated efforts include worldwide consortiums such as the International Cancer Genome Consortium (ICGC; icgc.org), The Cancer Genome Atlas (TCGA; cancergenome.nih.gov) and more recently the Genomic Data Commons (GDC; gdc.cancer.gov), which have collated data over multiple types of cancer on diverse molecular platforms, to accelerate discovery of molecular markers associated with cancer development and progression. The resulting analytical challenges are to integrate these vast amounts of data into models that accurately predict the complex pathophysiology of cancer and to translate this complexity into clinically actionable outputs, towards the holy grail of precision medicine.

Initial studies in cancer genomics relying on single platform analyses (mostly gene expression- and protein-based) have discovered multiple candidate “druggable” targets such as KRAS mutation in colon and lung cancer [1], BRAF in colorectal, thyroid, and melanoma cancers [2], and PI3K in breast, colon and ovarian cancers [3]. However, it is believed that integrating data across multiple molecular platforms has the potential to discover more co-ordinated changes on a global (unbiased) level [4]. Through data integration, we espouse the philosophy that cancer is driven by numerous molecular/genetic alterations and the interactions between them, with each type of alteration likely to provide a unique but complementary view of cancer progression. This offers a more holistic view of the genomic landscape of cancer, with increased power and lower false discovery rates in detecting important biomarkers [5], and translates to substantially improved understanding, clinical management and treatment [6].

Our methods are motivated by a TCGA based study in glioblastoma multiforme (GBM), where-in diverse platform-specific features are obtained at genomic, epigenomic and transcriptomic resolutions across matched tumor samples. Our goals are two-pronged: first assess dependence within and between platform-specific features, and second, incorporate the dependence in finding important molecular markers associated with relevant clinical outcomes. Integrating data across platforms has sound biological justifications due to interplay of features between and within the platforms. For example, between platforms, attributes at the genomic/DNA level such as methylation and copy number variation can directly affect mRNA expression, which in turn is known to influence clinical outcomes such as cancer progression times and pathobiology of the tumors. Within platform interactions arise from pathway-based dependencies (e.g. functional and signaling pathways) as well as dependencies based on chromosomal/genomic location (e.g. copy number data). Furthermore, the molecular features are inherently on different scales: discrete (copy number variation) and continuous (DNA methylation and mRNA expression). In addition, there exist substantial prior knowledge on pathway/graphical interactions between these genes (e.g. from public databases and literature), which can be incorporated to achieve improved estimation, increase signal to noise ratio and more refined biological interpretations. Our proposed approach combines all the above aspects to develop an integrative model for predicting clinical outcomes.

There has been a growing but limited literature on statistical and computational approaches exploiting the information garnered from data integration in relating the platforms to the clinical outcome—which is usually the goal of translational research in finding markers of cancer progression. Choi et al. [7] propose a double layered mixture model to jointly analyze copy number and gene expression data. Recently, Wang et al. [5] and Jennings et al. [8] proposed integrative Bayesian analysis of genomics data (iBAG, in short), which models biological relationships between genomic features from multiple platforms, and subsequently uses the estimated relationships to relate the platforms to a clinical outcome. However, iBAG assumes independence between genes in discovering mechanistic relationships between platforms at a gene-centric level, which may not be biologically practical as genes are known to lie on functional or cell signaling pathways [9].

Given that the associations between genes and gene products can be captured efficiently via networks, there is a growing variable selection literature for graph structured genomic covariates coming from a single platform [10]–[13] which account for the inherent dependencies in relating genetic biomarkers to the clinical outcome of interest. Such approaches either assume a known network structure on covariates (supervised), or estimate the graph from the raw data without considering prior knowledge (unsupervised). Both these classes of approaches have critical drawbacks. Supervised approaches may not be practical in genomic studies, since no existing and curated knowledge can be considered as complete and the gene network is likely to vary over different conditions, tumor types and biological processes. On the other hand, unsupervised approaches may often lead to inaccurate estimates because of the low signal to noise ratio [14], especially for high throughput genomic data typically collected on a low/moderate number of replicates. In these scenarios, there is an increasing recognition of the practical advantages of including prior biological knowledge when estimating gene networks from the data [15], which is not accounted for in existing structured variable selection approaches. Moreover to our knowledge, the existing structured variable selection approaches consider data from a single platform and are not equipped to handle mixed covariates from multiple platforms, which may give rise to different sets of between platform interactions not captured in a single platform analysis.

**Fig 1:**
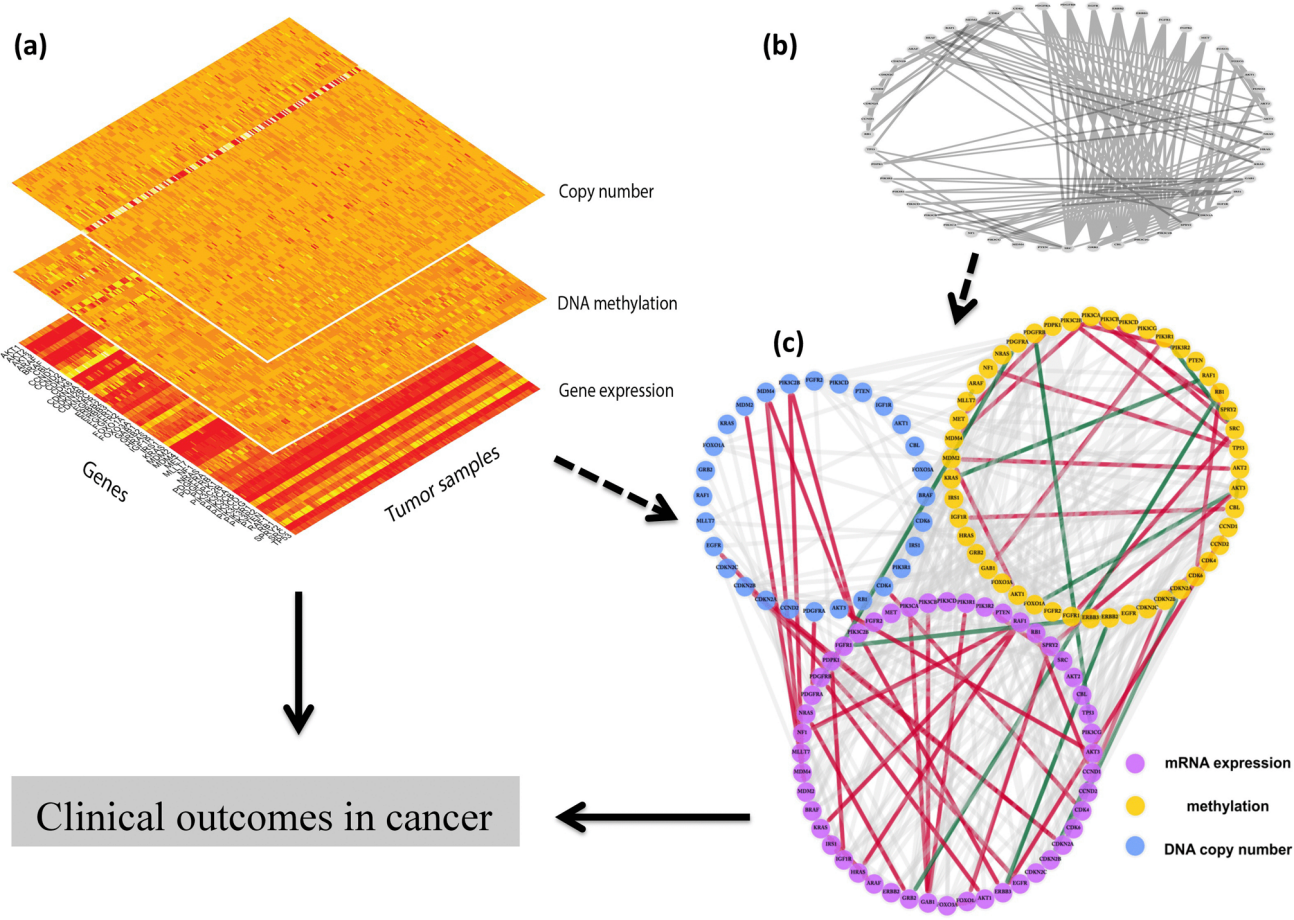
A schematic diagram of our integrative modeling approach. Panel (a) shows the heatmaps of the genes by sample matrix constructed from data for three platforms; panel (b) depicts the prior graph constructed using previous studies; while panel (c) is the estimated graph of the genes within and across the platforms. The dashed arrows determine graphical structure and the solid arrows represent the regression model incorporating graphical dependencies. Red and green lines in panel (c) represent high negative and positive partial correlations under the estimated graph, while all other edges with lower absolute partial correlations are depicted with watermark lines. We have also provided an interactive version of S1 Interactive Plot.

Unlike previous approaches incorporating prior information to estimate the graph based on single platform data ([16], [17]), the focus of our current work is to combine multi-platform and multi-scale genomic data, and prior knowledge on the gene network, to estimate the graph for mixed variables, and subsequently use structures in the estimated graph to inform variable selection via a novel clique based approach. In addition, the proposed network estimation approach involves a belief parameter to control the degree of fidelity to the prior knowledge in order to guard against mis-specification. Concisely stated, the major novelties of our approach are: (i) estimating graphical models for mixed data from multiple platforms, while incorporating prior graphical knowledge; (ii) developing a structured variable selection approach, which accounts for correlated groups of predictors within and across platforms, and can identify individual and groups of significant covariates related to the outcome (iii) allowing for both cis- and trans-acting relationships between molecular features, and providing appropriate controls for multicolleanarity and multiple testing. These goals are achieved via a principled multi-scale approach which involves a prior informed Bayesian graphical model for mixed variables in the first stage, which is then used to inform a subsequent Bayesian structured variable selection framework (see Fig 1). The above features make our approach distinct from existing structured variable selection approaches which typically focus on single platform data with known network knowledge [18].

## Methods

We focus on a univariate continuous response, *y* ∈ ℜ, to be regressed on a *p*-dimensional vector of mixed covariates **x** = [**x**_**1**_, …, **x**_D*_] obtained from D*(≥ 2) distinct platforms. However, we note that our approach can be generalized to binary or ordinal outcomes via latent variable based thresholding approaches [19], [20]. In our notations, **x**_*j*_ is the covariate vector corresponding to the j-th platform which has *p*_*j*_ features, *j* = 1, …, D*, so that $\sum_{j=1}^{D^{*}}p_{j}=p$. We note at the outset that the proposed approach allows for unequal number of measurements for different platforms, and hence it is possible to accommodate additional or missing measurements in one or more platforms. This is a useful feature, for example, when one has to include a methylation measurement that is far away from a gene but is highly relevant for its expression. Let us denote the *n* × 1 vector of responses as **y** and the *n* × *p* dimensional covariate matrix as *X* = [*X*_1_, …, *X*_D*_], where the covariates may be continuous, binary or ordered categorical. The mixed covariates have an underlying graphical structure which is to be estimated (e.g. panel (c) in Fig 1), while incorporating prior existing graphical knowledge, denoted by *G*_0_ (e.g. panel (b) in Fig 1). This is the graphical modeling or structure learning step, which is used to inform the subsequent structured variable selection step. The above steps comprise our two step approach which is described in detail below.

### First stage: Integrative structure learning

The graphical modeling approach for mixed data models ordered categorical variables by rounding continuous latent variables [19], [20], and specifies a graphical model jointly on the observed continuous covariates and the latent continuous variables. The graph for mixed data involves the vertex set *V* = {1, …, *p*} and edge set *E*, and is used to: (1) model dependence between features within and across platforms—in our application, measurements for different platforms are available for the same set of genes, so that the joint modeling across platforms allows for both *cis-acting* (localized to a gene) and *trans-acting* (across gene locations); and (2) detect potentially overlapping subgroups of features within and across platforms, which define functional modules that work together to drive the clinical outcome. Such modules correspond to cliques in the graph, which are defined as a subgroup of *V* such that each node in this subgroup is connected to every other node in the subgroup.

Without loss of generality, let $x_{i}=\left[x_{i1},\ldots ,x_{{\text{iD}}^{*}}\right]=\left(x_{i}^{C},x_{i}^{O}\right)$ denote the covariate vector for the i-th subject, with the superscripts *C*, *O* denoting continuous and ordinal (and/or binary) covariates respectively. Let *z*^*O*^ denote the generic notation for the latent continuous variable corresponding to ordinal predictor *x*^*O*^, and consider the following graphical model for mixed covariates

$$\begin{array}{ccc} x_{ij}^{O} & = & l,\;\text{if}\;D_{l-1}<=z_{ij}^{O}<D_{l},\;-\infty =D_{0}<D_{1}<\ldots <D_{M_{o}}=\infty ,\;j=1,\ldots ,p_{O},\\ z_{i}^{O} & = & \left(z_{i1}^{O},\ldots ,z_{ip_{O}}^{O}\right),\;\left(x_{i}^{C},z_{i}^{O}\right)∼N_{\left[D\right]}\left(0,\Omega^{-1}\right),\;\Omega ∼\pi \left(\Omega |G_{0}\right),\;i=1,\ldots ,n, \end{array}$$ (1)

where *N*_[*D*]_ denotes a Gaussian distribution with truncated domains defined by the hyper-rectangle *D*, *M*_*o*_ − 1 is the number of ordinal levels, *p*_*O*_ is the number of ordinal covariates, and Ω ∼ *π*(Ω∣*G*_0_) corresponds to a continuous shrinkage prior which depends on prior graph knowledge *G*_0_ (to be described in the sequel). Under the generic continuous shrinkage specification (1), the MCMC samples can be simulated from the posterior

$$\begin{array}{c} P\left(\Omega ,z_{1}^{O},\ldots ,z_{n}^{O}|X,G_{0}\right)\propto \pi \left(\Omega |G_{0}\right)\prod_{i=1}^{n}\{\prod_{j=1}^{p_{O}}\sum_{l=1}^{M_{o}}1\left(x_{ij}^{O}=l\right)1\left(D_{l-1}\leq z_{ij}^{O}<D_{l}\right)\}N\left(x_{i}^{C},z_{i}^{O};0;\Omega^{-1}\right). \end{array}$$

Subsequently a post-MCMC step can be implemented in order to obtain the graph estimate $\hat{G}$ by thresholding absolute partial correlations corresponding to the estimated precision matrix $\hat{\Omega }$, as elaborated in Section 3.

We use a continuous shrinkage prior on Ω as it enables us to update all elements of the precision matrix at every iteration, thus utilizing the full prior knowledge on all edges to drive inferences. We note that discrete mixture approaches [21] based on reversible jump Markov chain Monte Carlo may not be able to visit a sizable proportion of the edges even for moderate dimensional graphs under a finite number of Markov chain Monte Carlo runs. In such a case, these edges will not be updated at all, and will instead correspond to the initial choice of the adjacency matrix relying on the prior graph. Such an approach will not satisfy our objective of learning all possible edges of the graph from the data while incorporating prior knowledge, and hence we choose a continuous shrinkage approach over discrete mixture alternatives.

### Incorporating prior graph information

As mentioned before, there exists a huge amount of literature/databases describing the functional behaviors of genes, as characterized in metabolic, signaling and other regulation pathways. These include publicly available information on genes, biological pathways, Gene Ontology (GO) terms, gene-gene interaction networks e.g. Kyoto Encyclopedia of Genes and Genomes (KEGG) and Ingenuity Pathway Analysis (IPA) [22] or context-specific literature in various tumor types [23]. These sources can be queried to yield prior known connectivity graph between genes that can be brought into the network inference towards more biologically plausible structures.

Let *G*_0_ be the prior graph having vertex set *V* = {1, …, *p*} and edge set *E*_0_, with the corresponding adjacency matrix *A*_0_ = (*a*_0,*ij*_), where *a*_0,*ij*_ is the inclusion indicator for edge (*i*, *j*). Throughout this article, we consider undirected graphs so that *a*_0,*ij*_ = *a*_0,*ji*_ for all (*i*, *j*). We propose an approach which specifies an exponential prior on the diagonals and double exponential priors on the off-diagonal elements of Ω. Further, the shrinkage parameters are assigned a mixture distribution which incorporates prior knowledge. In particular, we have the following hierarchical formulation,

$$\begin{array}{c} \begin{array}{cc} \pi (\Omega & |\lambda ,p,\kappa ,G_{0})=\pi \left(\Omega |\lambda \right)\pi \left(\lambda |p,\kappa \right)\pi \left(p|\kappa ,G_{0}\right),\\ \pi (\Omega |\lambda ) & \propto \prod_{i=1}^{p}Exp\left(\omega_{ii};\lambda_{ii}\right)\prod_{i<j}DE\left(\omega_{ij};\lambda_{ij}\right)1\left(\Omega \in M^{+}\right),\\ \pi (\lambda_{ij}|p,\kappa ) & =\left(1-p_{ij}\right)Ga\left(\kappa_{ij}+a_{\lambda },b_{\lambda }\right)+p_{ij}Ga\left(a_{\lambda },b_{\lambda }\right),\\ \pi (p_{ij}|\kappa ,G_{0}) & =Be(a_{0,ij}\kappa_{ij}+a_{p},\left(1-a_{0,ij}\right)\kappa_{ij}+b_{p}),\;i\neq j,i,j=1,\ldots ,p, \end{array} \end{array}$$ (2)

where **p** = {*p*_*ij*_: *i* ≠ *j*, *i*, *j* = 1, …, *p*} are mixture weights, *M*^+^ is the set of positive definite matrices, **λ** is the vector of shrinkage parameters with dimension *p*(*p* + 1)/2, and *κ*_*ij*_ is the belief parameter for edge (i,j), for *i* ≠ *j* (*κ*_*ij*_ = *κ*_*ji*_ under an undirected graph). In (2), the shrinkage parameters **λ** shrinks the precision off-diagonals corresponding to absent edges towards zero, and is modulated by the prior graph information via the mixing proportions **p**. These mixing proportions are modulated by prior graph knowledge, and involve belief parameters which control the degree of fidelity to such knowledge.

Role of belief parameter: To understand the role of the belief parameter in prior specification, observe that *E*(*p*_*ij*_) = (*a*_0,*ij*_ *κ*_*ij*_ + *a*_*p*_)/(*κ*_*ij*_ + *a*_*p*_ + *b*_*p*_), which implies that for large *κ*_*ij*_ >> *b*_*p*_, *E*(*p*_*ij*_) ≈ 1 when *a*_0,*ij*_ = 1, and *E*(*p*_*ij*_) ≈ 0, when *a*_0,*ij*_ = 0. In extreme case when *κ*_*ij*_ → ∞, we have *p*_*ij*_ → 1 when *a*_0,*ij*_ = 1, and *p*_*ij*_ → 0 when *a*_0,*ij*_ = 0, which encourages small and large values of λ_*ij*_ respectively, for small values of *a*_λ_/*b*_λ_. This suggests that as *κ*_*ij*_ → ∞, the prior realizations of |*ω*_*ij*_| will be away from zero when *G*_0_ suggests the edge (*i*, *j*), and they will be very close to zero otherwise.

Through the use of a belief parameter, we can control the degree of confidence we place on the available prior graph information. This is a useful feature in enabling investigators to be flexible i.e. either skeptical or fairly confident about the prior knowledge, as the situation demands. In practice, we expect the belief parameter to be calibrated based on domain knowledge, by assigning large values of the belief when investigators are reasonably certain of the prior knowledge, and near zero values when such knowledge is absent or doubtful. For example, in many genomic applications (including ours), there is sufficient prior knowledge on within pathway interactions, but scant information about between pathway dependencies. When one is not sure about the choice of the belief parameter, we can let the data determine it’s value under a griddy Gibbs approach. More details about calibration of the belief parameter, as well as the griddy Gibbs approach, can be found in the sequel.

### Second stage: Regression and structured variable selection

In the second step, called structured variable selection, we incorporate the structural knowledge represented by the estimated graph $\hat{G}$ in regressing the outcome of interest on covariates. Although we consider continuous outcomes, it is straightforward to extend our approach to binary or ordinal outcomes via thresholding the latent continuous variables. We consider the following linear regression model

$$\begin{array}{ccc} y & = & \alpha 1_{n}+X_{\gamma }\beta_{\gamma }+\epsilon ,\;\epsilon_{i}∼N\left(0,\eta^{-1}\right),\;i=1,\ldots ,n,\\ \beta_{j} & ∼ & DE(\beta_{j};\eta_{1}),\;\gamma ∼\pi \left(\gamma |\hat{G}\right),j=1,\ldots ,p. \end{array}$$ (3)

Here ***γ*** = { *γ*_*j*_, *j* = 1, …, *p*} ∈ Γ (the model space) is the vector of variable inclusion indicators, with *γ*_*j*_ = 1 if the *j*th candidate predictor is included in the model and *γ*_*j*_ = 0 otherwise, ***β***_***γ***_ = {*β*_*j*_: *γ*_*j*_ = 1, *j* = 1, …, *p*} is the *p*_***γ***_ × 1 vector of the regression coefficients with $p_{\gamma }=\sum_{j=1}^{p}\gamma_{j}$ being the size of model ***γ***, *X*_***γ***_ is the *n* × *p*_***γ***_ covariate matrix (excluding an intercept) containing the predictors in model ***γ*** and having the i-th row as **x**_***γ***,*i*_. Further, we have $\alpha ∼N\left(\mu_{\alpha },\sigma_{\alpha }^{2}\right),\eta ∼Ga\left(a_{\eta },b_{\eta }\right),$ as the intercept and residual precision, respectively, while *η*_1_ is the shrinkage parameter for the double exponential (*DE*) prior on the fixed effects. We address uncertainty in subset selection through $\pi (\gamma |\hat{G})$ depending on the estimated graph structure on the covariates, while *π*(***β***_*j*_) characterizes the prior knowledge of the size of the coefficients for the *j*-th predictor, *j* = 1, …, *p*.

**Fig 2:**
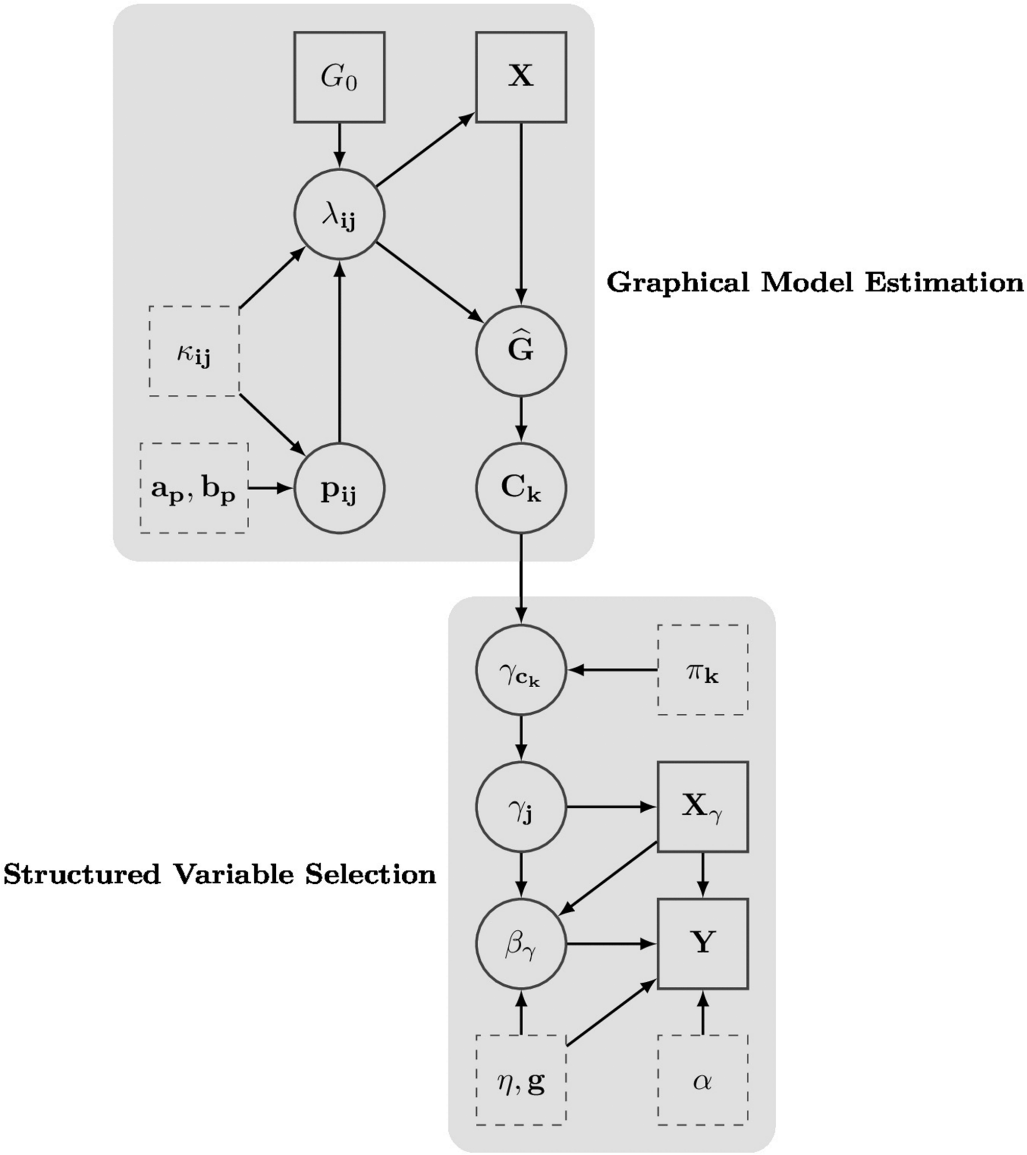
Directed acyclic graph of proposed model. The indices run: *i* ≠ *j* = 1, …, *p*, *k* = 1, …, *q*, with *q* being number of cliques in the estimated graph. Solid boxes, circles, dashed boxes, represent observed data, model parameters, and model hyperparameters respectively.

Priors on model space: The prior on the model space $\gamma ∼\pi (\gamma |\hat{G})$ is defined using clique indicators. Let *C*_1_, …, *C*_*q*_, denote the cliques identified by the estimated graph $\hat{G}$. The cliques are indicative of (potentially overlapping) groups of associated genetic features within and across platforms and gene locations. Denote the clique inclusion indicators as $\gamma_{C_{k}}$, *k* = 1, …, *q*, and let us define the prior on the model space as follows

$$\begin{array}{c} P\left(\gamma_{C_{k}}=1|\hat{G}\right)=\pi ,\;\pi ∼Be\left(a_{\pi },b_{\pi }\right), \end{array}$$ (4)

where *π* controls the sparsity of clique inclusions, under a multiplicity adjusted prior [24]. We call the resulting approach in (3) and (4) *Bayesian variable selection with structure learning* (BVS-SL), a schematic representation of which is presented in Fig 2. We note that when all the cliques are disjoint with *q* < *p*, the model loosely resembles a clustering approach allowing for different magnitudes of effects within a selected cluster. In the special case when *q* = *p*, our method reduces to the usual stochastic search variable selection (SSVS) approach, with Laplace priors on the fixed effects.

We focus on cliques as a building block in our structured variable selection approach, since (a) it is a systematic way of defining sub-groups of connected nodes in the graph which makes them intuitively appealing to work with, in structured variable selection problems involving correlated groups of predictors; and (b) cliques represent foundational blocks in a graph which have been used successfully in literature, to define probability distributions for Markov random fields under the Hammersley-Clifford Theorem [25], as well as to define likelihoods under decomposable graphs [21]. Although we focus on cliques, we note that the proposed approach can be generalized in a straightforward manner to alternate sub-groups of nodes having incomplete relationships. However, it is not immediately clear how one would define such incomplete subgroups in a manner which will facilitate variable selection, and this issue warrants a more thorough investigation.

Variable selection, clique identification and multiplicity controls: Variable selection proceeds by first identifying important cliques by computing the clique specific marginal inclusion probabilities. Individual significant covariates are then identified by including all covariates residing in significant cliques, and subsequently eliminating unimportant covariates which have near zero effect sizes from this set. This elimination proceeds via a post-MCMC approach which computes point-wise 95% credible intervals for *β*_*j*_ using MCMC iterations for which the *j*-th covariate was present in one or more significant cliques, and excluding all covariates for which the credible interval spans zero. This step, in addition to the multiplicity adjusted priors over cliques, enables control over false positives. Finally, non-zero effect sizes of all variables in a selected clique is expected to provide protection against collinearity for members within a clique. Thus, our approach is designed to attain a desirable balance between detecting true positives and true negatives, a claim which is supported by our simulation studies.

The variable selection approach espouses the philosophy that features in each clique are indicative of functional modules which work in coordination to drive the outcome. This assumption is somewhat similar to graph constrained penalized regression approaches [10] which assume that two neighboring genes in a network should be more likely to (or not to) participate in the same biological process simultaneously. These articles smooth the covariate effects for connected variables in the graph while encouraging sparsity and similar fixed effects, whereas no such assumptions are made under our approach. The proposed approach encourages covariates residing in important and unimportant cliques to be simultaneously included and excluded from the model respectively in order to tackle collinearity. However, the variables belonging to important cliques may not end up being included together in the final estimated model under the post-processing step which is designed to control false positives.

We examine if the inherent collinearity in the variables within a clique will potentially hamper the post-processing step desgined to exclude unimportant variables using 95% point-wise credible intervals. Letting ***γ*** denote the model involving all covariates belonging to at least one significant clique, the corresponding regression coefficients are drawn from the posterior distribution which is multivariate Gaussian with mean $A^{-1}X_{\gamma }^{T}\tilde{y}$ and covariance matrix *η*^−1^ *A*^−1^, where $A=(X_{\gamma }^{T}X_{\gamma }+\text{diag}\left(\tau_{1}^{2},\ldots ,\tau_{p}^{2}\right))$, and *τ* is the latent scale parameter under a scale mixture of Gaussians representation for the double exponential priors (see the posterior computation section for more details). Clearly, the posterior mean and variance are well-defined as long as ${\left(X_{\gamma }^{T}X_{\gamma }+\text{diag}\left(\tau_{1}^{2},\ldots ,\tau_{p}^{2}\right)\right)}^{-1}$ is non-singular, which is the case in most practical scenarios where the latent scale parameter values are learnt in a data driven manner. Note that the invertibility of the covariance matrix *A* is assured due to the term $\text{diag}(\tau_{1}^{2},\ldots ,\tau_{p}^{2})$ which adds to the diagonals of *X*^*T*^ *X*, ensuring positive definiteness. The above facts imply stable estimates for the variance for the estimated regression coefficients corresponding to the variables included via important cliques, which results in a successful elimination of false positives in the post-processing step, as evidenced in our extensive numerical studies.

### Posterior computation

The posterior computation for the proposed approach contains two independent sets of steps, corresponding to the two stages, as described below.

Computation for Graphical Model Estimation: The graphical model estimation for mixed covariates proceeds via sampling the latent underlying continuous variables corresponding to the ordered discrete covariates, followed by drawing the joint precision matrix of (**x**^*C*^, **z**^*O*^) under formulation (2). We adapt the procedure in Johnson and Albert (2001) to the case of dependent covariates, for posterior updates of the latent continuous covariates under the following posterior distributions

$$\begin{array}{c} z_{ij}^{O}\left|x_{ij}^{O}=l,D_{l-1,j},D_{l,j}∼N_{\left(D_{l-1,j},D_{l,j}\right]}\left(z_{j}^{O}|x_{i}^{C},z_{i}^{O}\left(-j\right)\right),\;D_{l,j}\right|z_{j}^{O}∼\text{Unif}\left(z_{l,j}^{L},z_{l,j}^{U}\right), \end{array}$$

where $z_{i}^{O}\left(-j\right)$ represents the vector of latent underlying variables for the i-th subject and excluding the j-th measurement, $z_{l,j}^{L}=\max_{i:x_{ij}^{O}=l}z_{ij}^{O}$ and $z_{l,j}^{U}=\min_{i:x_{ij}^{O}=l+1}z_{ij}^{O}$ for *l* = 1, …, *M* − 1. Once the latent variables have been updated at each MCMC iteration, we sample Ω using the method described in Wang [26], while λ_*ij*_ is updated using the posterior

$$\begin{array}{c} \pi \left(\lambda_{ij}|-\right)∼Ga(1+a_{\lambda },|\omega_{ij}|+b_{\lambda })1\left(\delta_{ij}=1\right)+Ga(1+\kappa_{ij}+a_{\lambda },|\omega_{ij}\left|+b_{\lambda }\right)1\left(\delta_{ij}=0\right), \end{array}$$

where 1(⋅) is an indicator function, *δ*_*ij*_ ∼ Bernoulli(*p*_*ij*_) and *p*_*ij*_ is drawn from a Beta posterior. Following Wang [26], the point estimate of the graph is obtained as a post–MCMC step by including the (i,j)-th edge if and only if ${\hat{\rho }}_{ij}/E_{W}\left(\rho_{ij}|X\right)>0.5$, where ${\hat{\rho }}_{ij}$ is the posterior partial correlation estimate under the continuous shrinkage approach, and $E_{W}\left(\rho_{ij}|X\right)$ represents the posterior mean of the partial correlation under the reference distribution $W=\text{Wishart}\left(3,I_{P}\right)$. Note that the belief parameter is either chosen *apriori* or it can be updated using a griddy Gibbs sampling step as well.

Computation for Structured Variable Selection: The computation strategy described above yields an estimate of the graph, which is used to inform the variable selection approach in the second step as described here. The Gibbs sampling alternates as follows

Step 1: Sample $\gamma_{C_{j}},j=1,\ldots ,p$, from Bernoulli($\pi_{j}^{+}$) posterior distributions where $\pi_{j}^{+}$ is the posterior inclusion probability for the j-th variable.
Step 2: Given ***γ***, sample the fixed effects ***β***_***γ***_ under a scale mixture of Gaussians representation for the double exponential distribution, as in [27].
Step 3: Sample the residual precision using $\pi (\eta |-)=Ga\left(n/2+a_{\eta },\sum_{i}{\left(Y^{n}-X_{\gamma }\beta_{\gamma }\right)}^{T}\left(Y^{n}-X_{\gamma }\beta_{\gamma }\right)/2+b_{\eta }\right)$.
Step 4: Letting q* = the number of cliques selected using Step 1, sample clique prior inclusion probabilities using *f*(*π*|−) = *Beta*(q* + *a*_*π*_, *q* − q* + *b*_*π*_).
Step 5: Sample *η*_1_ under the Gamma hyperpriors for $\eta_{1}^{2}$ as in [27].
Step 6: Sample the intercept *α* from a posterior distribution which is Gaussian.

Hyperparameter Choices: Below, we list the hyper-parameters used in the model, and elucidate the values we use for them, along with the justifications for such choices.

- The belief parameter *κ* is edge specific and chosen to have a high or low value according to whether we have high confidence on the prior graph knowledge or not. In the event where one is unsure about the level of confidence, a griddy Gibbs approach can be used, as outlined in S1 Appendix.
- We chose *ω*_*ii*_ ∼ *Exp*(*ω*_*ii*_∣λ_*ii*_), λ_*ii*_ ∼ *Ga*(10^−2^, 10^−6^), as recommended in the original Bayesian graphical lasso approach [26].
- Hyperparameters *a*_*p*_, *b*_*p*_, for *π*(*p*_*ij*_) in Eq (2) are chosen such that the ratio *a*_*p*_/*b*_*p*_ is small (we choose *a*_*p*_/*b*_*p*_ ≈ 0.1). This is because the prior mean for the edge inclusion probability is given by *E*(*p*_*ij*_) = (*a*_0,*ij*_ *κ*_*ij*_ + *a*_*p*_)/(*κ*_*ij*_ + *a*_*p*_ + *b*_*p*_), which implies that *E*(*p*_*ij*_∣*a*_0,*ij*_ = 1) ≈ 1, and *E*(*p*_*ij*_∣*a*_0,*ij*_ = 0) ≈ 0, for large values of the belief parameter, and a small value of *a*_*p*_/*b*_*p*_. In the case of no prior information (i.e. when *κ*_*ij*_ = 0,) we have *E*(*p*_*ij*_) = *a*_*p*_/(*a*_*p*_ + *b*_*p*_), which is small for small values of *a*_*p*_/*b*_*p*_, resulting in sparse graphs.
- We choose hyperparameters *a*_λ_, *b*_λ_ for *π*(λ) in Eq (2) such that *a*_λ_/*b*_λ_ is small. As explained previously, this encourages |*ω*_*ij*_| to be close to zero or away from it, depending on whether the prior information suggests the absence or presence of the corresponding edge.
- In Eq (3), we chose the prior on the residual precision as *η* ∼ *Ga*(0.1, 1) in the linear regression model, so as to encourage a residual distribution with thick tails corresponding to a non-informative prior which can accommodate large errors.
- The shrinkage parameter in the Laplace prior for the regression coefficients in Eq (3) is modeled under a conjugate Gamma distribution as $\eta_{1}^{2}∼Ga\left(1,2\right)$, which is close to the choice in the seminal Park and Casella (2008) [27] paper, and works well in a variety of simulation scenarios. The prior density is designed such that it approaches 0 sufficiently fast as $\eta_{1}^{2}\to \infty$ (to avoid mixing problems), and it is relatively flat and places high probability near the maximum likelihood estimate, as recommended in [27].
- We choose the hyperparameters *a*_*π*_ = 0.1, *b*_*π*_ = 1 for the prior on clique inclusion probabilities in Eq (4), to encourage a small number of cliques to be included in the regression model, which facilitates multiplicity control. This choice works well for controlling false positives for a wide variety of numerical experiments, in our experience.

## Results

### Simulation studies

We perform simulation studies to assess the variable selection and prediction performance for the proposed approach under several scenarios with varying dimensions and association structures for the covariates. The goal of our simulations is to examine the performance of our approach with existing unstructured variable selection approaches which do not take into account underlying structure information, by either assuming independence among covariates, or accounting for dependence in a way which is not tailored towards underlying network knowledge.

We implement the proposed approach both without and with prior knowledge corresponding to *κ* = 0 and *κ* = 50 respectively. In the first case, the graph is estimated completely from the data, and in the second case, the prior graph is taken to be the true graph *G*_0_ used to generate the data. The same value of the belief parameter (50) was used for all edges corresponding to strong confidence; however, we also examine the effect of varying the belief parameter as well as prior mis-specification as elaborated in S2 Appendix. We compare the proposed approach to stochastic search variable selection (SSVS) [28] assuming independence of predictors, the penalized joint credible regions approach (PenCred) by Bondell and Reich [29], and the spike and slab approach or SSL [30] which fuses the Bayesian spike and slab approach with elements of penalized likelihood estimation. We also compared the performance with penalized approaches such as Lasso [31], elastic net [32], and smoothly clipped absolute deviation or SCAD [33], using R packages ‘lars’, ‘elasticnet’ in CRAN, and ‘SSL’ in the authors’ website, respectively. The PenCred approach accounts for dependence within predictors, while the other approaches do not explicitly account for any such dependence but are they are widely used variable selection approaches. In addition, results were also included under a sparse fused lasso approach (Flasso) similar to the one described in [18], which encourages the coefficients of related features to share similar magnitudes under the penalized criteria $\frac{1}{2}\sum_{i=1}^{n}{\left(y_{i}-x_{i}\beta \right)}^{2}+\lambda \sum_{(i,j)\in E}|\beta_{i}-\beta_{j}|+\gamma \lambda \sum_{j=1}^{p}\left|\beta_{j}\right|$, where *γ* and λ are penalty parameters controlling the sparsity and the similarity between coefficients for connected variables in the graph, respectively, and *E* denotes the edge set in the given graph. This approach was implemented via the *fusedlasso* function in the *genlasso* package in R (https://cran.r-project.org/web/packages/genlasso/index.html). For the proposed approach, the cliques under the estimated graph $\hat{G}$ was determined via the ‘igraph’ package in R, and 10,000 MCMC iterations were run with a burn in of 3000. The training and test sample sizes were 100 each, and we consider *p* = 40, 80. All results are reported over 50 replicates.

For Cases I(a)-(d) stated below, the data was generated from a linear regression model having *p* covariates out of which nine were ordinal (generated by thresholding the continuous latent variables) and taking values 0-4, and the rest were continuous. The true inclusion status is set to $\gamma_{j}^{0}=1,j=1,\ldots ,8,23,24,$ with four discrete variables included, and $\gamma_{j}^{0}=0$ otherwise. The continuous covariates and the continuous latent variables for discrete covariates were generated using a multivariate Gaussian distribution with covariance Σ_*T*_. We consider different block-diagonal structures for Σ_*T*_ (listed hereafter), specifying subgroups of predictors with varying partial correlations. The true graph *G*_0_ which was used for BVS-SL with *κ* = 20, was obtained by including all edges (*i*, *j*) with $|\Sigma_{T}^{-1}\left(i,j\right)|>0.0001$.

**Case I(a):** This case corresponds to high partial correlations with the precision matrix having four sub-blocks and all precision diagonals being 1. The first sub-block (4 × 4) has off-diagonals as 0.95, the second and third sub-blocks (4 × 4 each) have precision off-diagonals as 0.7, and the fourth sub-block ($\bar{p-12}\times \bar{p-12}$) is identity. The true coefficient vector was (0.3, −0.7, 1.1, −0.05, 0.1, 0.2, −1.2, 1.5, 0, …, 0, 1, −1).
**Case I(b):** This case corresponds to high correlations with Σ_*T*_ having the same structure as $\Sigma_{T}^{-1}$ in Case I(a). The coefficients were (0.3, 0.7, 1.1, 0.05, −0.1, −0.2, −1.2, −1.5, 0, …, 0, 1, −1). Pair-wise positively correlated covariates have the same signs in both Case I(a) and I(b).
**Case I(c):** This case corresponds to a block diagonal with two sub-blocks—one having an AR(1) structure for the precision matrix with $\Sigma_{T}^{-1}\left(i,j\right)=0.95^{\left|i-j\right|},i,j=1,\ldots ,8,$ and the other sub-block being identity. The coefficients were same as those in Case I(a).
**Case I(d):** This case corresponds to Σ_*T*_ having the same structure as $\Sigma_{T}^{-1}$ in Case I(c). The coefficients were same as those in Case I(b).
**Case II:** We used the network for 48 genes in the TCGA data analysis to construct the inverse covariance matrix. In particular *p* was chosen as 48 × 2 and the inverse covariance matrix has a block diagonal form with sparse associations across two equally sized sub-blocks of dimension 48 × 48 each, and the associations within each sub-block being determined by the gene network information provided in [34]. Data was generated from a Gaussian graphical model having the true coefficient vector as (0.3, −0.7, 1.1, −0.05, 0.1, 0.2, −1.2, 1.5, **0**_38_, 1, −1, 0.3, −0.7, 1.1, −0.05, 0.1, 0.2, −1.2, 1.5, **0**_38_, 1, −1), where **0**_38_ is a vector of zeros of dimension 38, which resembles the coefficient vector in Case I(a).

Cases I(a)-(d) capture the different simulation scenarios with distinct platforms, where measurements within platforms are captured via an auto-regressive structure or they are uncorrelated, and there are no connections across platforms. The unequal sized sub-blocks represent measurements which are available on only one platform but are not available on others. On the other hand, Case II resembles the TCGA data example with two equally sized platforms, where the associations within each platform is determined via prior network knowledge [34] and there being sparse associations across platforms.

**Fig 3:**
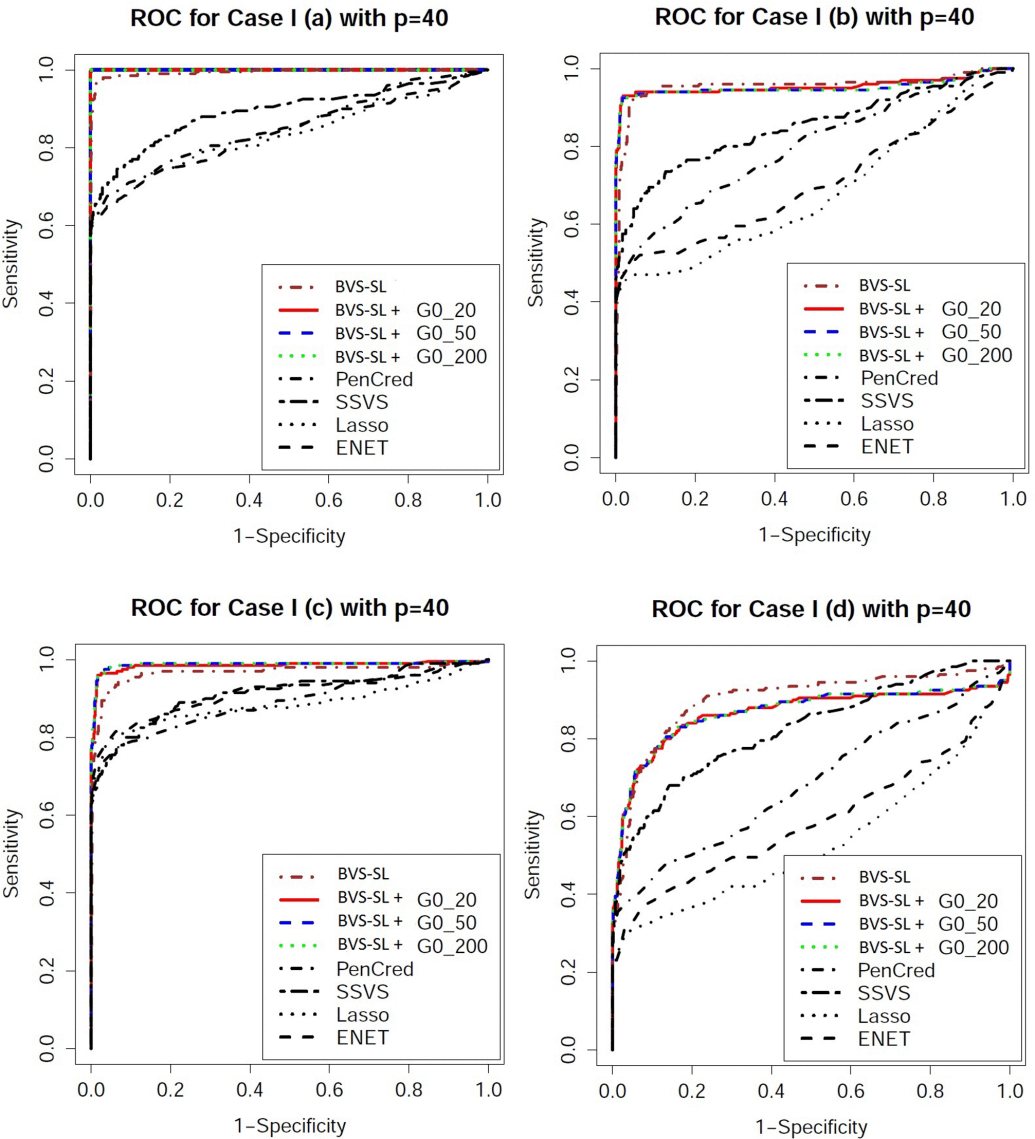
Receiver operating characteristic plots for *p* = 40 under Models 1(a)-(d). BVS-SL + *G*0_*κ* represents the Bayes variable selection with structure learning with belief parameter *κ* for all edges. Pencred, SSVS, Lasso, ENET, represent the penalized credible regions approach, stochastic search variable selection, *L*_1_ penalized regression, and elastic net, respectively. The curves for SSL and SCAD are not presented to ensure greater clarity of the plot.

**Fig 4:**
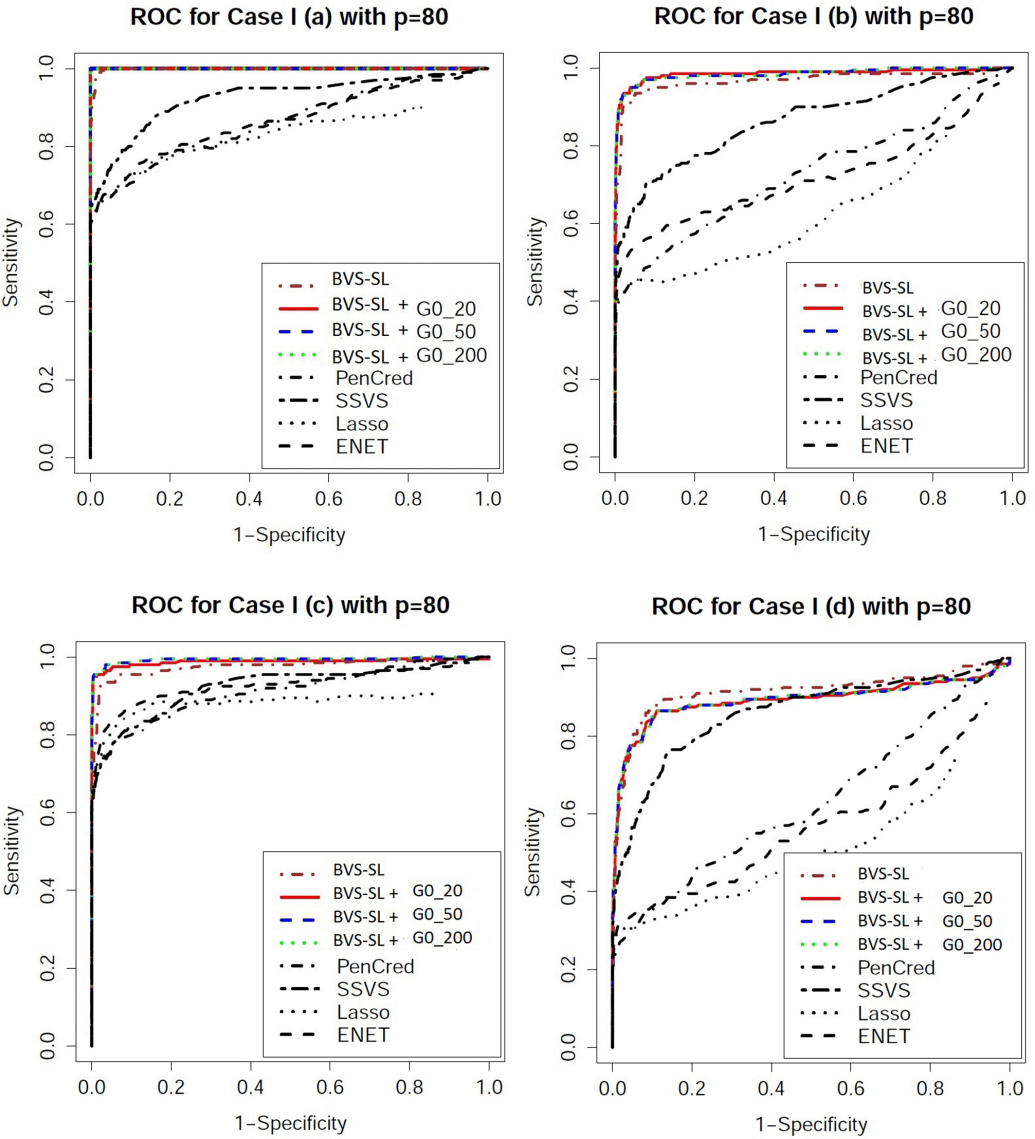
Receiver operating characteristic plots for *p* = 80 under Models 1(a)-(d). BVS-SL + *G*0_*κ* represents the Bayes variable selection with structure learning with belief parameter *κ* for all edges. Pencred, SSVS, Lasso, ENET, represent the penalized credible regions approach, stochastic search variable selection, *L*_1_ penalized regression, and elastic net, respectively. The curves for SSL and SCAD are not presented to ensure greater clarity of the plot.

**Fig 5:**
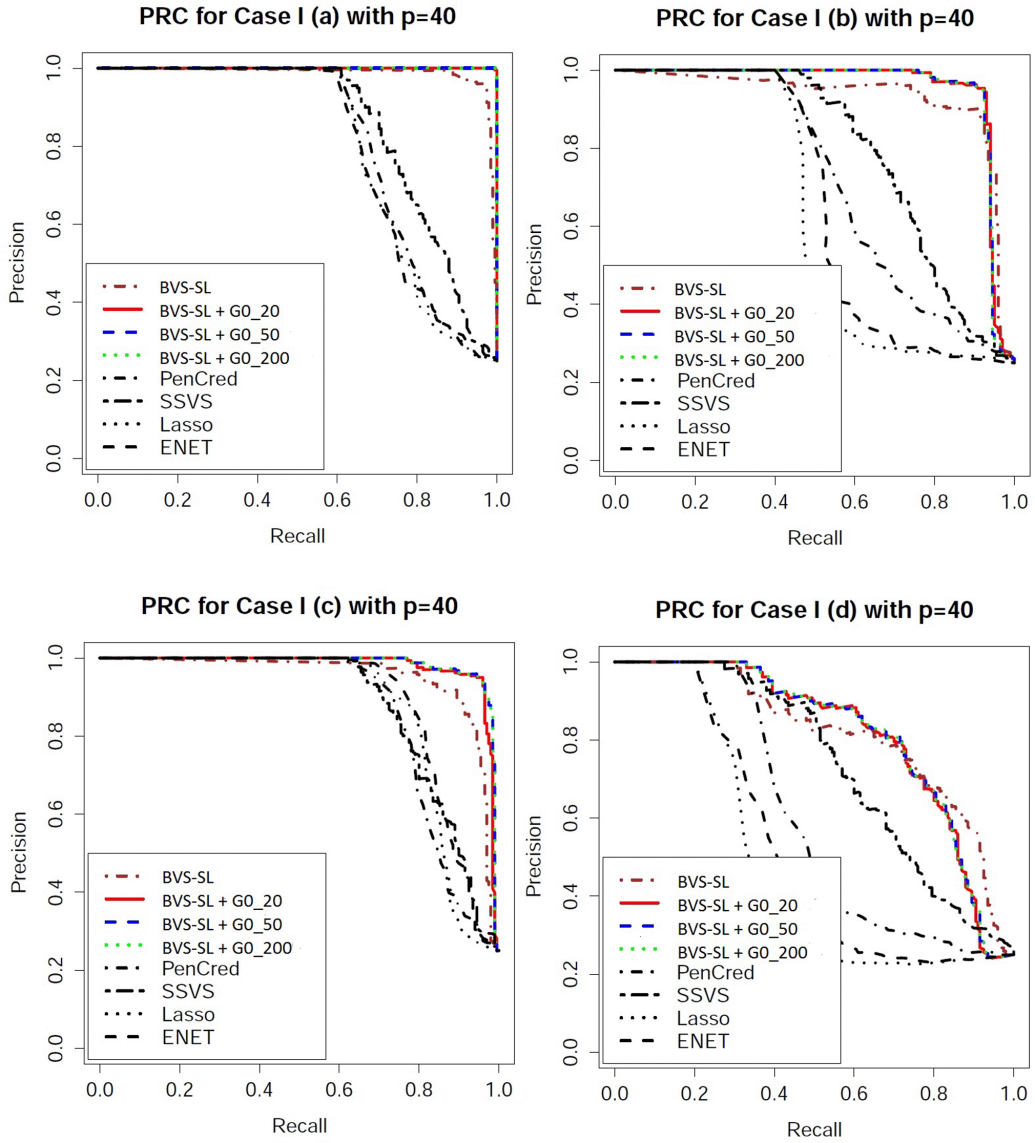
Precision recall characteristic plots for *p* = 40 under Models 1(a)-(d). BVS-SL + *G*0_*κ* represents the Bayes variable selection with structure learning with belief parameter *κ* for all edges. Pencred, SSVS, Lasso, ENET, represent the penalized credible regions approach, stochastic search variable selection, *L*_1_ penalized regression, and elastic net, respectively. The curves for SSL and SCAD are not presented to ensure greater clarity of the plot.

**Fig 6:**
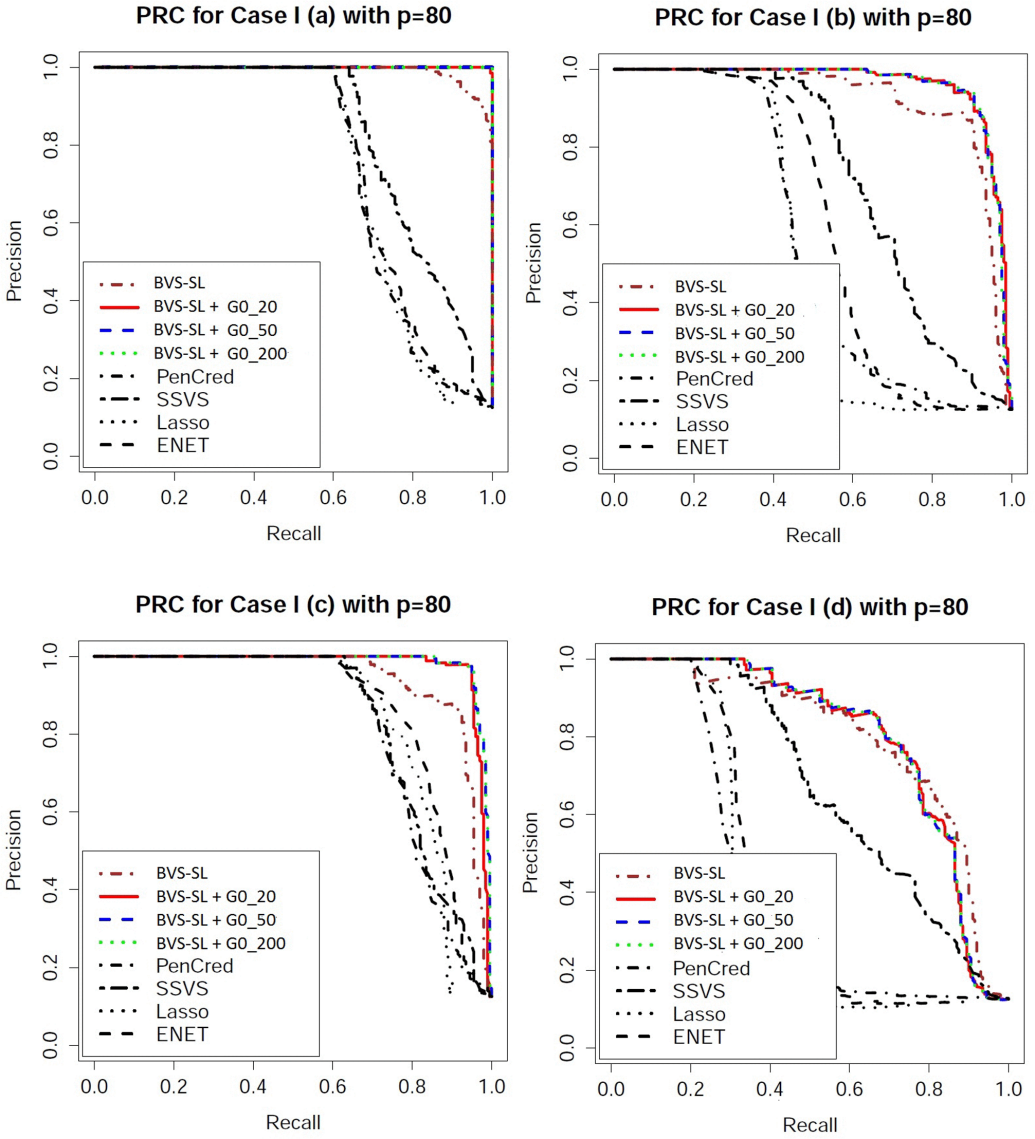
Precision recall characteristic plots for *p* = 80 under Models 1(a)-(d). BVS-SL + *G*0_*κ* represents the Bayes variable selection with structure learning with belief parameter *κ* for all edges. Pencred, SSVS, Lasso, ENET, represent the penalized credible regions approach, stochastic search variable selection, *L*_1_ penalized regression, and elastic net, respectively. The curves for SSL and SCAD are not presented to ensure greater clarity of the plot.

Performance evaluation: One can obtain an ordered sequence of regression models by varying the cut-off for the marginal inclusion probability under Bayesian approaches and varying the penalty parameter for frequentist approaches. To evaluate the ordering of the models, we look at receiver operating characteristic (ROC) curves which plot the sensitivity versus 1-specificity, and precision recall characteristic (PRC) curves which plot the precision (ratio of true positives to the total number declared as positive) versus sensitivity. From the ROC and PRC curves presented in Figs 3–6, it is clear that BVS-SL with and without prior graph knowledge essentially always dominate the competing curves, while also having a significantly higher area under the curve as shown in columns 2 and 3 in Tables 1–4. Moreover, BVS-SL demonstrates a significantly and uniformly higher power when the false discovery rate is controlled at 10%, which points towards a superior performance in tackling collinearity for a given multiplicity level, as shown in column 4 in Tables 1–4.

**Table 1:** Simulations for Case I(a)-I(b), training sample size = 100, test sample size = 100. BVS-SL(*κ*) represents the Bayes variable selection with belief parameter *κ* for all edges. Pencred, SSVS, Lasso, EL, SCAD, SSL, and Flasso represent the penalized joint credible regions approach, stochastic search variable selection, *L*_1_ penalized regression, and elastic net, the smooth clipped absolute deviation, the spike and slab lasso, and sparse fused lasso respectively. MSPE: out of sample predictive MSE; Pwr(10% FDR) is sensitivity controlling for 90% specificity; MS: estimated model size; FP: false positives, and *Cov*_95_ is coverage under 95% predictive intervals. The true model size for Cases I(a)-(b) is 10.

Method	MSPE	ROC	PRC	Pwr(10% FDR)	MS	FP	*Cov*_95_
**Case I(a) p = 40**							
BVS-SL(*κ* = 0)	1.103	0.997	0.996	**1.000**	10.000	0.444	**0.922**
BVS-SL(*κ* = 20)	**1.100**	**1.000**	**1.000**	**1.000**	10.250	0.250	0.921
PenCred	1.153	0.890	0.869	0.802	9.400	1.700	0.912
SSVS	1.123	0.954	0.921	0.880	7.650	**0.200**	0.917
Lasso	1.206	0.894	0.870	0.815	10.950	2.900	0.893
EL	1.218	0.906	0.874	0.815	11.150	3.050	0.893
SCAD	1.310	0.707	0.714	0.824	9.64	1.72	0.915
SSL	1.309	0.855	0.825	0.762	7.36	0.46	0.908
Flasso	1.228	0.912	0.894	0.851	9.258	0.623	0.895
**Case I(a) p = 80**							
BVS-SL(*κ* = 0)	1.093	0.999	0.996	**1.000**	9.800	**0.250**	0.928
BVS-SL(*κ* = 20)	**1.092**	**1.000**	**1.000**	**1.000**	10.600	0.600	**0.930**
PenCred	1.270	0.888	0.790	0.740	13.700	6.150	0.880
SSVS	1.128	0.965	0.897	0.895	8.050	0.750	0.926
Lasso	1.288	0.879	0.770	0.800	10.700	3.100	0.883
EL	1.300	0.890	0.773	0.790	11.050	3.450	0.879
SCAD	1.370	0.697	0.680	0.746	9.12	1.70	0.905
SSL	1.364	0.887	0.834	0.732	7.08	0.36	0.908
FLasso	1.155	0.938	0.912	0.854	9.190	0.428	0.904
**Case I(b) p = 40**							
BVS-SL(*κ* = 0)	1.108	0.971	0.947	0.955	9.100	1.000	0.924
BVS-SL(*κ* = 20)	1.111	**0.987**	**0.981**	**0.985**	9.550	0.650	0.923
PenCred	1.156	0.749	0.679	0.550	6.200	1.200	0.912
SSVS	**1.107**	0.842	0.785	0.695	6.400	0.650	**0.925**
Lasso	1.251	0.630	0.563	0.423	8.650	4.050	0.885
EL	1.265	0.648	0.598	0.465	8.300	3.450	0.881
SCAD	1.287	0.664	0.632	0.662	8.64	1.78	0.912
SSL	1.274	0.831	0.792	0.652	6.34	**0.28**	0.914
Flasso	1.114	0.851	0.827	0.792	9.59	0.90	0.898
**Case I(b) p = 80**							
BVS-SL(*κ* = 0)	1.089	0.960	0.927	0.950	8.200	0.500	0.922
BVS-SL(*κ* = 20)	**1.082**	**0.975**	**0.963**	**0.965**	8.600	0.400	**0.927**
PenCred	1.212	0.738	0.561	0.518	9.450	4.450	0.888
SSVS	1.093	0.863	0.730	0.725	6.050	0.600	0.924
Lasso	1.290	0.609	0.473	0.450	6.100	2.200	0.861
EL	1.295	0.628	0.536	0.530	7.250	2.750	0.861
SCAD	1.408	0.655	0.599	0.658	10.80	4.06	0.901
SSL	1.305	0.816	0.740	0.632	6.28	**0.18**	0.912
Flasso	1.182	0.885	0.851	0.802	9.24	0.73	0.901

**Table 2:** Simulations for Cases I(c)-(d) and Case II, training sample size = 100, test sample size = 100. BVS-SL(*κ*) represents the Bayes variable selection with belief parameter *κ* for all edges. Pencred, SSVS, Lasso, EL, SCAD, SSL, and Flasso represent the penalized joint credible regions approach, stochastic search variable selection, *L*_1_ penalized regression, and elastic net, the smooth clipped absolute deviation, the spike and slab lasso, and sparse fused lasso respectively. MSPE: out of sample predictive MSE; Pwr(10% FDR) is sensitivity controlling for 90% specificity; MS: estimated model size; FP: false positives, and *Cov*_95_ is coverage under 95% predictive intervals. The true model size for Cases I(a)-(b) is 10.

Method	MSPE	ROC	PRC	Pwr(10% FDR)	MS	FP	*Cov*_95_
**Case I(c) p = 40**							
BVS-SL(*κ* = 0)	1.071	0.969	0.929	0.965	7.941	0.529	**0.936**
BVS-SL(*κ* = 20)	**1.066**	**0.986**	**0.978**	**0.980**	8.000	**0.100**	**0.936**
PenCred	1.118	0.879	0.860	0.780	9.150	1.750	0.921
SSVS	1.086	0.880	0.894	0.825	7.150	0.250	0.932
Lasso	1.142	0.857	0.787	0.808	11.550	3.550	0.918
EL	1.145	0.895	0.847	0.820	11.250	2.950	0.917
SCAD	1.338	0.615	0.579	0.646	0.822	1.84	0.914
SSL	1.240	0.866	0.830	0.638	6.34	0.22	0.927
FLasso	1.128	0.917	0.894	0.903	8.68	0.610	0.924
**Case I(c) p = 80**							
BVS-SL(*κ* = 0)	1.092	0.951	0.927	0.935	8.000	0.316	**0.928**
BVS-SL(*κ* = 20)	**1.084**	**0.992**	**0.988**	**0.990**	7.650	**0.050**	**0.928**
PenCred	1.253	0.867	0.770	0.735	12.650	5.750	0.889
SSVS	1.109	0.895	0.796	0.745	6.600	0.200	0.921
Lasso	1.179	0.814	0.729	0.695	10.650	3.800	0.909
EL	1.189	0.861	0.770	0.738	9.900	3.100	0.910
SCAD	1.343	0.644	0.637	0.658	7.16	0.70	0.901
SSL	1.330	0.867	0.789	0.654	6.22	0.08	0.908
FLasso	1.193	0.892	0.886	0.814	8.92	0.250	0.906
**Case I(d) p = 40**							
BVS-SL(*κ* = 0)	1.072	**0.922**	0.851	**0.845**	6.850	0.850	0.934
BVS-SL(*κ* = 20)	**1.065**	0.893	**0.857**	0.825	6.250	0.400	**0.935**
PenCred	1.126	0.691	0.622	0.480	6.450	1.700	0.915
SSVS	1.071	0.855	0.767	0.645	5.500	0.500	0.933
Lasso	1.218	0.556	0.475	0.340	5.000	1.750	0.891
EL	1.225	0.594	0.516	0.390	5.100	1.550	0.893
SCAD	1.262	0.653	0.599	0.568	7.90	1.78	0.921
SSL	1.247	0.792	0.742	0.590	5.74	**0.14**	0.924
Flasso	1.118	0.863	0.838	0.0.791	7.46	0.842	0.902
**Case I(d) p = 80**							
BVS-SL(*κ* = 0)	1.109	**0.937**	0.788	**0.865**	5.900	0.900	**0.919**
BVS-SL(*κ* = 20)	**1.105**	0.894	**0.798**	0.815	5.800	**0.400**	0.918
PenCred	1.226	0.633	0.419	0.358	7.850	4.150	0.897
SSVS	1.124	0.840	0.655	0.628	4.600	**0.400**	0.914
Lasso	1.239	0.509	0.354	0.320	4.650	1.700	0.886
EL	1.248	0.596	0.406	0.355	4.800	1.700	0.881
SCAD	1.488	0.619	0.517	0.448	12.78	6.60	0.892
SSL	1.253	0.787	0.683	0.574	5.82	**0.400**	0.917
FLasso	1.182	0.849	0.722	0.694	6.81	0.88	0.912

**Table 3:** Simulations for Case II, training sample size = 100, test sample size = 100. BVS-SL(*κ*) represents the Bayes variable selection with belief parameter *κ* for all edges. Pencred, SSVS, Lasso, EL, SCAD, SSL, and Flasso represent the penalized joint credible regions approach, stochastic search variable selection, *L*_1_ penalized regression, and elastic net, the smooth clipped absolute deviation, the spike and slab lasso, and sparse fused lasso respectively. MSPE: out of sample predictive MSE; Pwr(10% FDR) is sensitivity controlling for 90% specificity; MS: estimated model size; FP: false positives, and *Cov*_95_ is coverage under 95% predictive intervals. The true model size is 10.

Method	MSPE	ROC	PRC	Pwr(10% FDR)	MS	FP	*Cov*_95_
Case II							
BVS-SL(*κ* = 0)	1.26	**0.89**	0.79	**0.78**	17.85	1.55	0.901
BVS-SL(*κ* = 20)	**1.15**	0.86	**0.83**	0.72	17.10	0.80	**0.912**
PenCred	1.22	0.66	0.55	0.42	17.45	2.10	0.880
SSVS	1.30	0.81	0.72	0.69	15.90	**0.75**	0.905
Lasso	1.38	0.59	0.47	0.39	13.90	2.85	0.892
EL	1.39	0.63	0.58	0.40	14.10	2.55	0.895
SCAD	1.38	0.63	0.59	0.59	12.72	2.5	0.901
SSL	1.32	0.78	0.73	0.68	10.72	1.70	0.866
FLasso	1.22	0.83	0.81	0.70	15.10	1.05	0.896

**Table 4:** Graphical model estimation performance of the proposed method (BVS-SL) approach under different values of the belief parameter, in the case where the mixed covariates consist of continuous and binary variables, where the binary predictors are generated as in Eq (5) under logit and probit links. The results for different precision matrices structures as in Cases I(a)-(d), are presented in terms of specificity and sensitivity under the estimated graph, and the error in estimating the precision matrix in terms of the Frobenius norm.

Method	Link	Sensitivity	Specificity	Fnorm
Case I(a)				
BVS-SL(*κ* = 0)	logit	1.00	0.778	3.678
BVS-SL(*κ* = 50)	logit	0.98	1.00	2.024
BVS-SL(*κ* = 0)	probit	1.00	0.775	3.678
BVS-SL(*κ* = 50)	probit	0.99	1.00	1.877
Case I(b)				
BVS-SL(*κ* = 0)	logit	0.99	0.79	3.711
BVS-SL(*κ* = 50)	logit	0.96	1.00	1.979
BVS-SL(*κ* = 0)	probit	0.99	0.79	3.687
BVS-SL(*κ* = 50)	probit	0.97	1.00	1.912
Case I(c)				
BVS-SL(*κ* = 0)	logit	0.60	0.78	5.98
BVS-SL(*κ* = 50)	logit	0.58	1.00	5.24
BVS-SL(*κ* = 0)	probit	0.59	0.78	6.06
BVS-SL(*κ* = 50)	probit	0.56	1.00	5.34
Case I(d)				
BVS-SL(*κ* = 0)	logit	0.63	0.76	5.87
BVS-SL(*κ* = 50)	logit	0.57	1.00	5.23
BVS-SL(*κ* = 0)	probit	0.66	0.77	5.78
BVS-SL(*κ* = 50)	probit	0.56	1.00	5.14

Prediction and Variable Selection: In addition to looking at the ordered sequence, we also investigated the predictive performance of each approach, as well as to assess the point estimate under the optimal model. The point estimate is selected using the Bayesian information criterion under PenCred, Lasso, and elastic net, while the median probability rule along with subsequent thresholding (using credible intervals) is used for BVS-SL, and the median probability rule is used for SSVS. We report the model size (MS) and false positives (FP) under the point estimate. This point estimate is also used for prediction under PenCred, Lasso, and elastic net, while the posterior predictive distribution is used under BVS-SL and SSVS. We look at the predictive performance in terms of out of sample mean squared error (RMSPE) and out of sample coverage of 95% predictive intervals (COV_95_). The coverage refers to the proportion of test sample values contained within predictive intervals. The predictive intervals correspond to credible intervals for the Bayesian approaches BVS-SL and SSVS, whereas for PenCred, as well as the frequentist approaches, they correspond to pseudo confidence intervals that are constructed as $\left(x\hat{\beta }-1.96\sigma_{0},x\hat{\beta }+1.96\sigma_{0}\right)$, where *σ*_0_ is the true residual variance.

It is seen from the first and last columns in Tables 1–4 that the proposed approach has superior performance in terms of out of sample prediction and 95% coverage with respect to competitors for almost all cases. The number of true covariates (MS—FP) detected under the proposed approach, as well as the coverage, is essentially always the best or the second best among all the approaches considered. We also see from the second last column that while the SSVS may have an advantage compared to BVS-SL with no structural information in terms of controlling false positives, the BVS-SL with *κ* = 20 essentially has similar or better multiplicity control compared to SSVS, thus demonstrating the advantages of incorporating prior information. On the other hand, the SSVS demonstrates drawbacks in terms of collinearity, as evidenced by smaller model sizes, and poor power to detect true positives for a given level of false discovery. Moreover, it is also evident that the SSL approach may have a lower FP rate in some cases; however this is possibly due overly sparse models reported by SSL (evident from the small model sizes) which can also result in a poor overall performance under the method. Finally, we note that the fused lasso approach may result in some improvements in variable selection performance over alternate approaches not incorporating prior knowledge, but it essentially always has less accurate performance compared to the proposed method. Moreover, the predictive performance under the fused lasso approach may not be optimal and even lower than generic approaches assuming independence between predictors. In summary, it is clear that the proposed approach seems to perform well both in terms of variable selection and prediction, while simultaneously tackling the conflicting issues of collinearity and multiplicity in the presence of correlated predictors.

Sensitivity to link function: In order to examine the performance of our approach when the link function which is used to relate the latent underlying variable to the discrete variables is mis-specified, we performed additional experiments where the ordinal variables in Cases I(a)-(d) were replaced with binary variables generated via a logit link. In particular, we modified model (1) as follows

$$\begin{array}{ccc} x_{ij}^{O} & = & I\left(z_{ij}^{O}>0\right),\;\left(z_{i}^{O},x_{i}^{C}\right)∼N_{\left[D\right]}\left(0,\Omega_{T}^{-1}\right),\;\Omega_{T}∼\pi \left(\Omega_{T}|G_{0}\right),\;i=1,\ldots ,n, \end{array}$$ (5)

where

$$\begin{array}{c} \Omega_{T}=[\begin{array}{cc} {\tilde{\sigma }}^{2}\phi_{i}^{-1}\Omega^{O} & \Omega_{12}^{O}\\ \Omega_{21}^{O} & \Omega_{22} \end{array}], \end{array}$$

and $\phi_{i}∼Ga\left(\tilde{\nu }/2,\tilde{\nu }/2\right)$, ${\tilde{\sigma }}^{2}=\pi^{2}\left(\tilde{\nu }-2\right)/\left(3\tilde{\nu }\right)$, with $\tilde{\nu }=7.3$. We note that $var\left(z_{ij}^{O}\right)={\tilde{\sigma }}^{2}\phi_{i}^{-1}$ and the off-diagonal elements of Ω_*T*_ encode within and between platform interactions. The latent variables *z*^*O*^ are thresholded at zero to yield binary predictors which marginally follow a logistic distribution. We considered different structures for Ω_*T*_ similar to Cases I(a)-(d), incorporating prior graph information *G*_0_ on the mixed covariates via the inverse covariance matrix. The true inclusion status is set to $\gamma_{j}^{0}=1,j=1,\ldots ,8,23,24,$ with four binary variables included, and $\gamma_{j}^{0}=0$ otherwise.

We examine the graph estimation performance of our method when the mixed covariates are generated as above, and compare these results with the scenario when a probit link is used to generate the latent variables which can be implemented by setting ${\tilde{\sigma }}^{2}=\phi_{i}^{-1}=1$ in (5). The results presented in Table 4 clearly suggest that (a) irrespective of the link used to generate the binary variables, a higher value of the belief parameter results in better graphical model estimation performance; and (b) the sensitivity and the specificity of the estimated graphs are very similar under both the links, even though there may be possible differences in the precision matrix estimation accuracy. Based on the above findings, we conclude that there are no systematic differences in terms of graphical model estimation, when the latent variables are generated under different links, which illustrates the robustness of the proposed approach.

### Integrative network analysis of TCGA glioblastoma data

Our motivating dataset arises from a TCGA-based study in glioblastoma multiforme (GBM), which is the most common and aggressive form of primary brain cancer in human adults. The TCGA data portal provides multiple levels of molecular data for a large cohort of GBM tumor specimens. Each qualified specimen was assayed using multiple assays among which we concentrate on the following: messenger RNA (mRNA) expression using HT-HG-U133A (Affymetrix) arrays, DNA methylation (METH) using HumanMethylation27K (Illumina) and DNA copy number (CN) HG-CGH-244A (Agilent) arrays. All the resulting data from the three platforms are pre-processed, normalized and annotated to the gene level. We focus our analysis on 48 genes that overlap with the three critical signaling pathways—RTK/PI3K, p53, and Rb, which are involved in migration, survival and apoptosis progression of cell cycles in cancer [23]. These pathways are dominantly dis-regulated in GBMs, as confirmed by integrative analyses of TCGA GBM samples [35]. Furthermore, the activity of these pathways is seen to vary across molecular subtypes, suggesting potential for therapeutic targeting (via inhibition of receptor tyrosine kinase activity) and prognostic assessment [23]. Thus reconstructing the topology and connectivity of these genes and pathways and evaluating the downstream impact on GBM prognostic time can shed light into the underlying cellular and biological mechanisms involved in the evolution of the GBM disease process. Thus our covariate matrix consists of 48 genes mapped to these core pathways from D* = 3 platforms (mRNA, METH, CN) resulting in *p* = 48 × 3 = 144 regressors. Note that mRNA and METH are continuous, while CN is discrete having three categories corresponding to loss, gain, or neutral. The outcome is log-transformed survival times for 233 patients which is regressed on the covariates using an accelerated failure time model. Among 233 patients, 70 were censored, whose survival times were imputed using Kaplan-Meier imputation.

Prior knowledge: The prior knowledge on the graphical structure between these 48 genes is based on previous studies in GBM [34], and is denoted as *G*_0,*pr*_ (shown in panel (b) of Fig 1). This prior graph is obtained by assessing sequence mutations, copy number alterations and proteins and confirm and extend the observation that GBM alterations tend to occur within specific functional modules. The prior graph in our analysis comprises 144 nodes, across the 3 platforms, and is constructed so as to preserve the prior graphical structure *G*_0,*pr*_ within the platforms, while allowing the data to infer interactions between two different platforms. Thus the prior graph can be concisely written as: *G*_0_ = *G*_0,*pr*_ ⊗ *I*_3_, where ⊗ represents the Kronecker product of the two matrices. Since we have strong prior knowledge about within platform interactions, we choose a high value for the belief parameter (*κ* = 50) within platforms. However we are unsure of the between platform associations and hence we choose a near zero *κ* value corresponding to these interactions, so that we learn these interactions directly from the raw data without imposing a strict prior belief.

**Fig 7:**
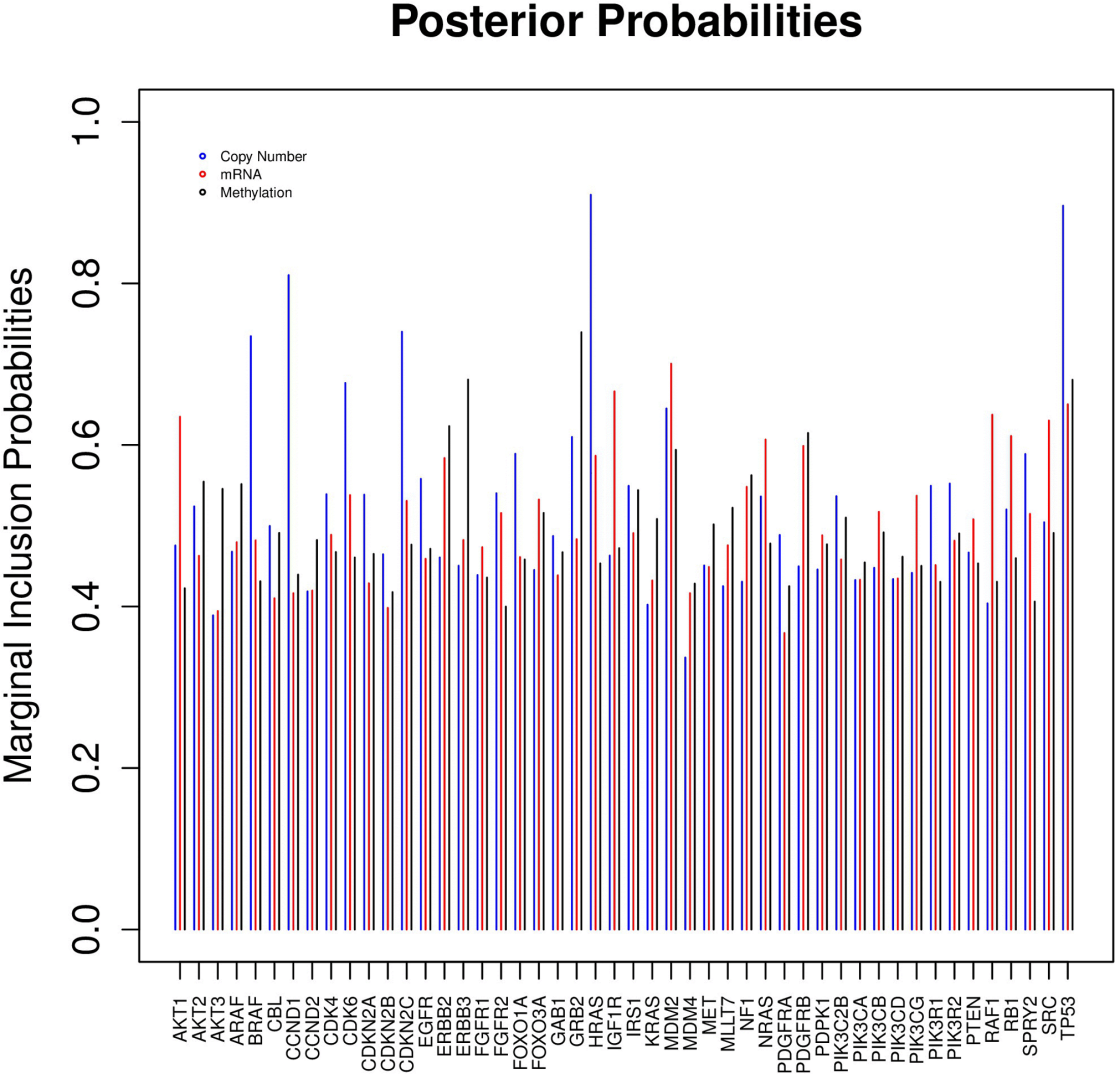
Marginal inclusion probabilities for each gene over three platforms. The probabilities are presented for the three platforms grouped by genes, with blue, red and black, implying copy number, mRNA expression, and methylation, respectively.

Results for survival-time association: We first surveyed the main prognostic (multi-platform) markers that were associated with the survival time of the GBM patients. The marginal inclusion probabilities of the variables using our analysis are presented in Table 5, with a corresponding plot in Fig 7 in the manuscript. We select the posterior probability threshold to infer important features under a false discovery rate criteria controlled at a pre-specified level, similar to the method described in [36]. In particular, we can choose a threshold *ϕ*_*θ*_ for posterior probabilities so as to control the average Bayesian FDR at level *θ*, which essentially implies that we expect 100*θ*% of the significant markers to be false positives. To obtain such an estimate, first sort the posterior probabilities for all markers in ascending order to yield *pr*_(*j*)_, *j* = 1, …, *p*. Then *ϕ*_*θ*_ = *pr*_(*ζ*)_, where $\zeta =\max\{j^{*}:j^{*-1}\sum_{j=1}^{j^{*}}pr_{\left(\zeta \right)}\leq \theta \}$.

**Table 5:** Analysis results for integrative genomics application for features having marginal inclusion probability greater than 0.5. MIP is marginal inclusion probability, and degree is the number of edges for a particular node.

Gene (platform)	MIP	Effect size	Degree	Gene name	MIP	Effect size	Degree
HRAS(CN)	0.91	-1.22	1	AKT2(METH)	0.55	0.04	4
TP53(CN)	0.89	-0.39	1	PIK3R2(CN)	0.55	-0.09	1
CCND1(CN)	0.81	-0.37	1	ARAF(METH)	0.55	0.004	2
CDKN2C(CN)	0.74	0.22	2	IRS1(CN)	0.54	0.13	2
GRB2(METH)	0.73	0.07	9	PIK3R1(CN)	0.54	0.04	2
BRAF(CN)	0.73	-0.13	3	NF1(mRNA)	0.54	0.01	8
MDM2(mRNA)	0.71	-0.07	7	AKT3(METH)	0.54	0.04	15
ERBB3(METH)	0.68	-0.07	9	IRS1(METH)	0.544	-0.03	8
TP53(METH)	0.68	0.14	5	FGFR2(CN)	0.54	0.05	8
CDK6(CN)	0.67	0.09	4	CDK4(CN)	0.53	-0.02	5
IGF1R(mRNA)	0.66	-0.07	10	CDKN2A(CN)	0.53	-0.07	7
TP53(mRNA)	0.65	0.07	5	CDK6(mRNA)	0.53	0.01	7
MDM2(CN)	0.64	-0.01	2	PIK3CG(mRNA)	0.53	-0.04	6
RAF1(mRNA)	0.63	-0.09	12	PIK3C2B(CN)	0.53	0.04	4
AKT1(mRNA)	0.63	-0.08	6	NRAS(CN)	0.53	0.1	1
SRC(mRNA)	0.63	0.06	6	FOXO3A(mRNA)	0.53	0.03	7
ERBB2(METH)	0.62	-0.07	15	CDKN2C(mRNA)	0.53	0.03	11
PDGFRB(METH)	0.61	-0.06	11	AKT2(CN)	0.52	-0.07	1
RB1(mRNA)	0.61	0.06	8	MLLT7(METH)	0.522	-0.04	3
GRB2(CN)	0.60	0.14	2	RB1(CN)	0.52	-0.03	4
NRAS(mRNA)	0.60	0.06	9	PIK3CB(mRNA)	0.51	-0.03	5
PDGFRB(mRNA)	0.59	0.05	6	FOXO3A(METH)	0.51	0.01	12
MDM2(METH)	0.59	-0.06	14	FGFR2(mRNA)	0.51	-0.01	3
FOXO1A(CN)	0.58	0.07	5	SPRY2(mRNA)	0.51	0.01	11
SPRY2(CN)	0.58	0.1	1	PIK3C2B(METH)	0.51	-0.05	6
HRAS(mRNA)	0.58	-0.05	11	KRAS(METH)	0.50	-0.05	5
ERBB2(mRNA)	0.58	0.04	6	PTEN(mRNA)	0.50	0.03	5
NF1(METH)	0.56	-0.04	4	SRC(CN)	0.50	-0.06	1
EGFR(CN)	0.55	0.07	5	MET(METH)	0.50	-0.01	9

Under a level 0.2 (corresponding to a posterior probability threshold of 0.7), and after thresholding, seven genes are significantly associated with progression through various mechanisms. They are (a) HRAS, TP53, CCND1, BRAF and CDKN2C, through copy number, (b) GRB2 through methylation, and (c) MDM2 through mRNA. Of these CDKN2C and GRB2 are positive drivers of progression, while the remaining genes are negatively associated with progression. HRAS is a member of the RAS oncogene family, whose negative effect on Glioblastoma is previously observed on the overall and progression-free survival [37]. CCND1 belongs to the Cyclin D family of cell cycle regulators, which are known to be up-regulated and amplified in malignant glioma [38]. Similarly, MDM2 the inhibitor of the tumor suppressor TP53, is established to be a candidate gene associated with short progression [39]. TP53 copy-number itself is associated with poor progression of GBM via deletion [40]. Although, there is no evidence of BRAF amplification in GBM, a previous study established that BRAF amplification via gene duplication event activates the MAPK signaling in low-grade glioma [41]. Moreover, CDKN2C is a well characterized tumor suppressor gene associated with many cancers and known to be deleted in Glioblastoma [42]. On the other hand, GRB2 is a key protein in epidermal growth factor receptor signaling in the Glioblastoma tumoroginesis pathway [43].

Clique analysis: The important cliques are identified ass those which have significant marginal clique inclusion probabilities. The clique analysis depicted multiple interesting two-way interactions. In certain cases, the multiple cliques containing the same molecular probe but with different partners have highly significant marginal inclusion probabilities. For instance AKT1 (METH) clique interaction with many different molecular probes is significant (Table 6). These cliques constitute both tumor suppressing as well as activating interactions. The cliques involving AKT1 (METH), PTEN (mRNA) and AKT1 (METH), PIK3R2 (mRNA) can be construed as tumor suppressing, while cliques involving AKT1 (METH), CCND1 (mRNA) and AKT1 (METH), GRB2 (CN) probably are tumor activating. The diverse biological functionality of the cliques represent the inherent biological subtypes within GBM [44].

**Table 6:** Cliques containing AKT1(METH). MIP stands for marginal inclusion probability.

Clique members		MIP	Clique members		MIP
AKT1(METH)	CCND1(mRNA)	0.86	CDKN2A(METH)	AKT1(METH)	0.69
AKT1(METH)	PTEN(mRNA)	0.84	AKT1(METH)	TP53(mRNA)	0.68
AKT1(METH)	PIK3R2(mRNA)	0.81	AKT1(METH)	RAF1(mRNA)	0.68
AKT1(METH)	GRB2(cn)	0.71	AKT2(METH)	AKT1(METH)	0.67

**Fig 8:**
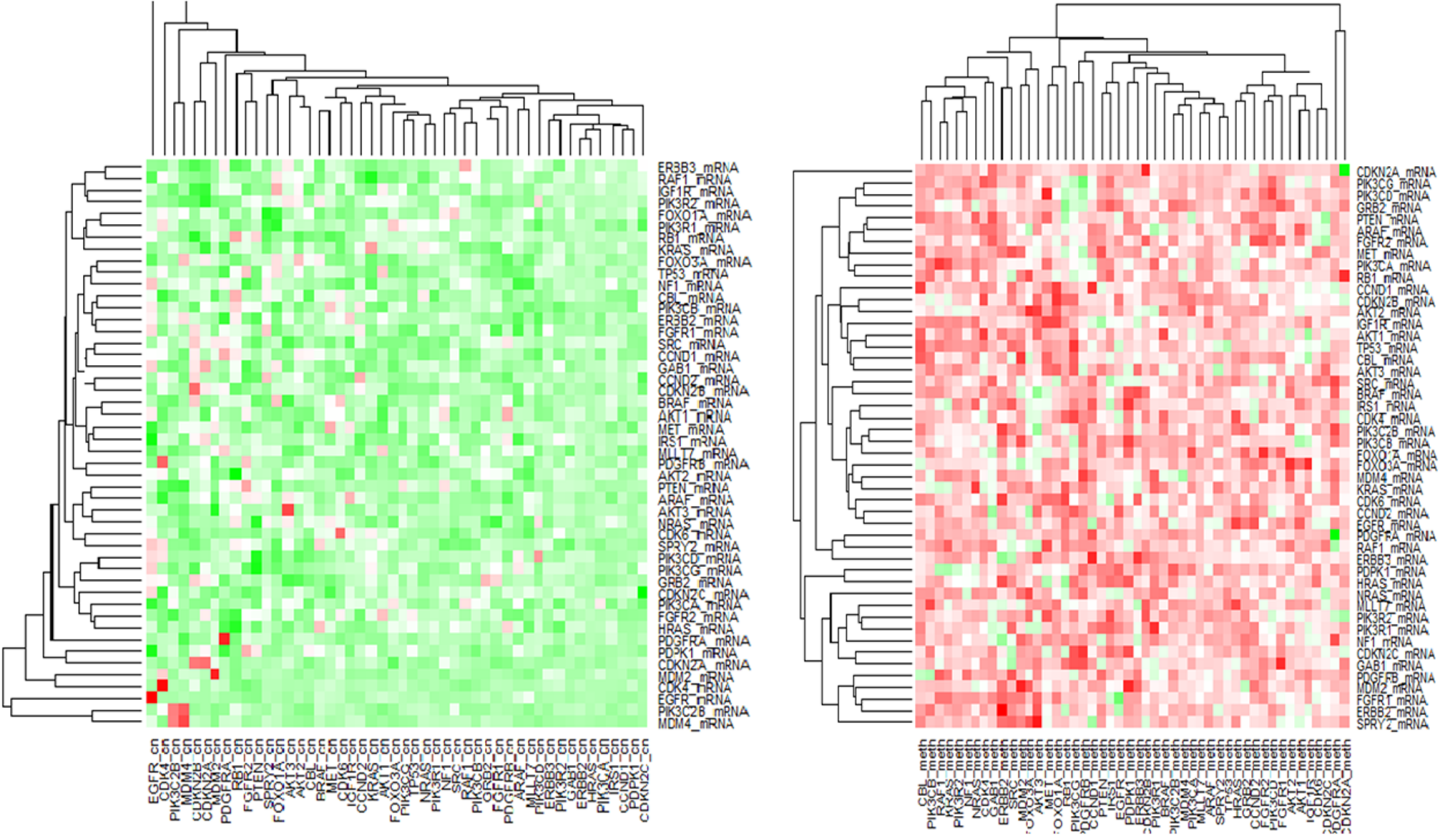
Left heatmap: Hierarchical clustering of correlation between the mRNA and copy number data; right heatmap: Hierarchical clustering of correlation between the mRNA and DNA methylation data. Green and red pertains to positive and negative partial correlations respectively.

Neighborhood analysis: In addition to detecting important prognostic markers for GBM, we also examine the estimated graph (panel (c) of Fig 1) within and across platforms. We take a closer look at the neighborhood of GRB2, which plays a central biological role in this molecular network as a trigger of the RAS signaling upon the activation of upstream receptor tyrosine kinase family members. The presence of three important tumor suppressor genes of GBM in the neighborhood of GRB2 (RB1, CDKN2B and PIK3CG) is interesting, although they have no direct interaction with GRB2. RB1 and PIK3CG seem to lose their functionality through DNA methylation, while CDKN2B through copy number loss, enabling the RTK/RAS activation cascade via GRB2. These events reinforce the previous illustration in GBM that hypermethylation and deletion of RB1 and CDKN2B respectively contribute to the loss of tumor suppressor function [45]. The partial correlations of genes between the platforms is demonstrated via clustering heatmaps in Fig 8. From the Figure, it is clear that there is a enrichment of positive correlations between the mRNA and copy number data, and an enrichment of negative correlations between the mRNA and DNA methylation data, which further supports our biological–hypothesis driven integrative models.

We performed additional data analysis where (a) no graph information was used (*κ* = 0); and (b) only 75% confidence was placed on the prior graph knowledge for within platform interactions, which was implemented by setting (*κ* + *a*_*p*_)/(*κ* + *a*_*p*_ + *b*_*p*_) = 0.75. The results are presented in detail in S2 Appendix and point to considerable overlap between the different analyses results.

## Discussion

We propose a novel multi-scale Bayesian structured variable selection approach, which is equipped to simultaneously learn the graphical structure from mixed-scale data sources in the presence of prior knowledge, and subsequently uses such structure learning to inform variable selection in a manner that controls for collinearity and multiple testing. In this paper, we focused on integrating (more upstream) copy number, mRNA expression and methylation markers associated with cancer progression; a future task is to extend our methods to account for downstream post-transcription and translational events such microRNA and proteomics markers. This will provide vital clues towards understanding the complete genomic landscape of cancer development and progression. Although we consider a cancer genomics application in this paper, we note that the application of this work is very general and can be applied to any regression setting with heterogeneous covariates lying on a graph.

In this paper, we worked with Level 3 TCGA data, where all the genomic platforms have been *a priori* matched at the gene-level which was used in all our downstream analyses. While this suffices for most genomic platforms, it might be useful to look at the more granular intra-genic correlations e.g. CpG sites for DNA methylation and SNPs for copy number data. For example, for methylation data we can choose the CpG islands within the gene body as well as the “shores” (say +/- few kilobase pairs outside the gene body) that might be better representative of the methylation profile for a gene, especially in assessing associations with other genomic platforms e.g. expression [46]. However, this would increase our covariate space and graphical model parameters considerably, given multiple CpG sites per gene. In addition, we can use the genomic locations for inform our prior edge calibration parameter—that we presume might induce some sparsity. We leave this task for future consideration.

The proposed approach could be further improved by accounting for non-linear graphical connections, as well as non-linear relationships between the outcome and predictors. Although the proposed method relies on cliques in the estimated graphical structure to account for collinearity, the approach can be generalized more incomplete connections between variables if it is possible to define such subgroups in a meaningful manner. Moreover, in practical applications where tumor heterogeneity is present, it is reasonable to expect subgroups of subjects corresponding to different but unknown tumor types to have different gene networks. In such cases, the proposed approach needs to be extended to unsupervised clustering approach incorporating a distinct gene network for each cluster. In addition, another potential improvement would be to propose a more efficient computational strategy which allows for greater scalability in terms of the number of covariates, which would enable us to construct genome-wide networks. In summary, network science is a rapidly evolving field with the main focus on the exploration of structural properties and dynamical behaviors of complex networked systems [47]–[49], and the proposed approach makes an important and timely contribution to this research area.

## Supporting information

S1 Interactive Plot Interactive version of Fig 1. We have generated an additional interactive pdf figure containing subpanels (a) and (c) in Fig 1, which enables to reader to zoom in a look at these panels of the diagram in greater detail.
(PDF)

S1 Appendix Calibration of the belief parameter. This Appendix contains guidance on the choice of the belief parameter based on the degree of confidence that one would like to put on the prior belief.
(PDF)

S2 Appendix Sensitivity to prior knowledge. This Appendix contains further results on the sensitivity of the BVS-SL approach when the mis-specification of the prior knowledge is varied in simulations. It further contains separate analysis of the TCGA data when (a) no graph information was used (*κ* = 0); and (b) only 75% confidence was placed on the prior graph knowledge for within platform interactions.
(PDF)

S1 Data TCGA data used in the article. The data file is a. Rdata file which contains the survival time and censoring status for subjects, as well as the copy number variation, gene expression and methylation measurements of the probes matched to the 48 genes considered in the real data analysis.
(7Z) Click here for additional data file.
