# Supplementary figures and images for: Bayesian variable selection with graphical structure learning: Applications in integrative genomics
Source: PLoS One. 2018 Jul 30;13(7):e0195070. doi: 10.1371/journal.pone.0195070 (PMC6066211; doi:10.1371/journal.pone.0195070)

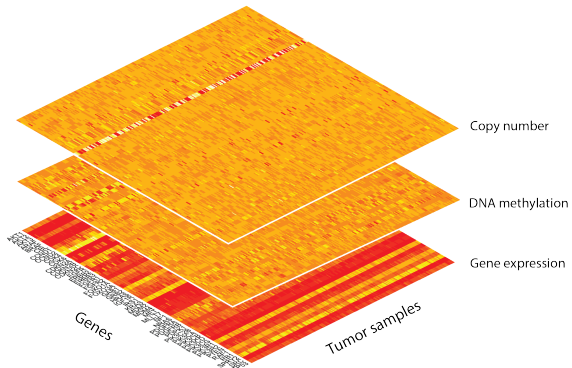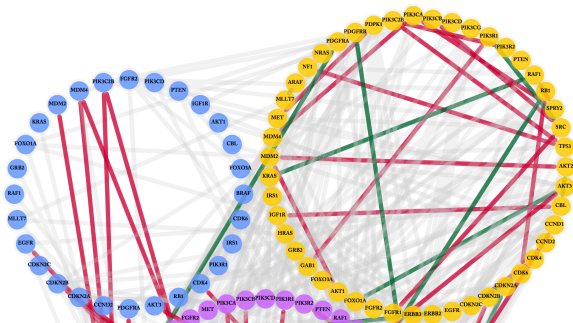

Supplement: S1 Interactive Plot — We have generated an additional interactive pdf figure containing subpanels (a) and (c) in Fig 1, which enables to reader to zoom in a look at these panels of the diagram in greater detail. (PDF) [file pone.0195070.s001.pdf]
