# Supplementary material for: Bayesian variable selection with graphical structure learning: Applications in integrative genomics
Source: PLoS One. 2018 Jul 30;13(7):e0195070. doi: 10.1371/journal.pone.0195070 (PMC6066211; doi:10.1371/journal.pone.0195070)
Supplement: S1 Appendix — This Appendix contains guidance on the choice of the belief parameter based on the degree of confidence that one would like to put on the prior belief. (PDF) [file pone.0195070.s002.pdf]

S1 Appendix: Calibration of the Belief Parameter

In practice we can choose $\kappa_{ij} >> b_p$ to quantify the degree of confidence on the presence of an edge specified by prior knowledge using

$$E(p_{ij}) = (a_{0,ij}\kappa_{ij} + a_p)/(\kappa_{ij} + a_p + b_p),\ E(\lambda_{ij}) = (1 - p_{ij})(\kappa_{ij} + a_\lambda)/b_\lambda + p_{ij}a_\lambda/b_\lambda, \quad (1)$$

so that $E(p_{ij} \mid a_{0,ij} = 1) \approx 1$, and $E(p_{ij} \mid a_{0,ij} = 0) \approx 0$. On rearranging the terms in (1) one has

$$\kappa_{ij} = b_p/(1 - E(p_{ij})) - (a_p + b_p),\ \text{when } a_{0,ij} = 1, \quad \kappa_{ij} = a_p/E(p_{ij}) - (a_p + b_p),\ \text{when } a_{0,ij} = 0.$$

Thus, if G_0 suggests presence of the edge (i, j), and we are 50% sure of the correctness of such prior knowledge, we can substitute $E(p_{ij}) = 0.5$ in the above expression to obtain $\kappa_{ij} = b_p - a_p$. In practice, one can obtain different values for the belief parameter corresponding to various values for $E(p_{ij})$. Fig S1 presents a plot of the belief parameter versus the degree of confidence for the scenarios when the prior graph suggests presence ($a_{0,ij} = 1$) and absence ($a_{0,ij} = 0$) of the (i,j)-th edge. From the plot, one can categorize different regions of confidence. For example, when $a_{0,ij} = 1$, a low confidence may correspond to $E(p_{ij}) < 0.5(-1 \leq \kappa_{ij} < 0)$, moderate confidence may correspond to $0.5 \leq E(p_{ij}) \leq 0.7(0 \leq \kappa_{ij} \leq 1.34)$, and high confidence may correspond to $E(p_{ij}) > 0.7(\kappa_{ij} > 1.34)$. We note that these cut-offs would depend on the problem at hand and one can choose different cut-off values which is more appropriate for a particular application. Similar regions can also be constructed for $a_{0,ij} = 0$.

In practice, we typically expect a sufficiently strong prior knowledge about the presence or absence of an edge corresponding to a reasonable proportion of connections within platforms, gleaned from existing databases, as well as the structural/mechanistic inter-platform interactions. For edges with reliable prior knowledge, one can specify a high value of the belief parameter, while for other edges corresponding to minimal or dubious prior information, one would specify a zero value of the belief parameter, which results in unsupervised learning of these edges.

For multiple within-platform connections, another possibility is quantifying knowledge from multiple databases (e.g. historical, literature based) where prior associations are implicated. These include standard databases such as KEGG, Biocarta, String DB (for proteins), as well as more recent developments such as experimentally-validated disease-specific databases such as Ontology Fingerprints for cancer [**?**]. In such cases, one can compute a semantic similarity score e.g. proportion of times a particular gene-gene relationship (edge) is present across these databases, and treat this score as the prior probability for an edge (or a group of edges). In this case, one can set $(\kappa + a_p)/(\kappa + a_p + b_p)$ as the semantic similarity score as outlined in (1), using which the value of the belief parameter can be derived.

When one is not sure about the choice for the belief parameter for certain edges, it can be updated under a data driven manner using a griddy Gibbs algorithm. This enables the data to determine the fidelity to the prior knowledge. Let $E_{0,\kappa}$ be the set of edges for which one is not sure about the choice of the belief parameter. From S1 Fig, we see that a value of $\kappa = 50$ is sufficient to ensure a high confidence on the prior belief. Hence we propose to use a fine grid of values for the belief parameter in the range $\kappa \in (-1, 50)$ for the griddy Gibbs approach, which uses the following form of the posterior

$$\begin{aligned}\pi(\kappa \mid -) \quad &\propto \quad \{\prod_{(i,j)\in E_{0,\kappa}} \frac{1}{(\kappa + a_\lambda)!}\lambda_{ij}^{\kappa+a_\lambda-1}(\frac{1}{b_\lambda})^{\kappa+a_\lambda}\}^{I(a_{0,ij}=1)} \\ &\times \quad \{\prod_{(i,j)\in E_{0,\kappa}} \frac{1}{Be(a_{0,ij}\kappa + a_p, (1 - a_{0,ij})\kappa + b_p)} p_{ij}^{a_{0,ij}\kappa+a_p-1}(1 - p_{ij})^{(1-a_{0,ij})\kappa+b_p-1}\},\end{aligned}$$

Fig S1. Calibration of belief parameter. The plot shows the range of the the belief parameter (y-axis) versus degree of confidence (x-axis). The three bins demarcated by vertical blue lines depict regions of low ($-1 \leq \kappa < 0$), moderate ($0 \leq \kappa < 1.34$) and high confidence ($\kappa > 1.34$) from left to right.

where κ is the common value of the belief parameter for all edges in $E_{0,\kappa}$, and $I(\cdot)$ is the indicator function. We can use a multinomial distribution to sample the belief parameter after normalizing the above posterior probabilities.
