# Supplementary material for: Bayesian variable selection with graphical structure learning: Applications in integrative genomics
Source: PLoS One. 2018 Jul 30;13(7):e0195070. doi: 10.1371/journal.pone.0195070 (PMC6066211; doi:10.1371/journal.pone.0195070)
Supplement: S2 Appendix — This Appendix contains further results on the sensitivity of the BVS-SL approach when the mis-specification of the prior knowledge is varied in simulations. It further contains separate analysis of the TCGA data when (a) no graph information was used (κ = 0); and (b) only 75% confidence was placed on the prior graph knowledge for within platform interactions. (PDF) [file pone.0195070.s003.pdf]

S2 Appendix: Sensitivity to prior knowledge:

Simulated Data: We performed additional simulation studies, where the prior graph had $10\%, 20\%, 50\%$ and 90% wrong edges, in order to assess the performance of our method under prior mis-specification. We present the results in terms of the area under the ROC and PRC curves under Cases I(a)-I(d) and for $p = 40$ in the Table 1. When compared to other approaches such as PenCred, SSVS, Lasso and elastic net, the area under the curve for the proposed method is higher corresponding to a high value of the belief parameter when there is no mis-specification, but the relative performance under the proposed approach may deteriorate when the proportion of mis-specified edges increases. In particular for $p = 40$, the area under the ROC and/or the PRC curve for the proposed approach when $\kappa = 50$ is lower than competing approaches when the proportion of incorrectly specified edges is 0.50 under Cases I(c) and I(d), but the corresponding area under the curve could be lower even when the proportion of mis-specified edges is 0.25, under Cases I(a) and I(b) . However, the area under the ROC and PRC curves is higher under the proposed approach for all cases when 10% of the edges are mis-specified, for $\kappa = 50$. Moreover, in the case where the working graph knowledge is fully correct, S2 Fig illustrates that the area under the ROC and PRC curves for case I(b) gradually increases with the belief parameter, before tapering off. On the other hand, the area under the curve for a low value of the belief parameter is more robust to mis-specification, and degrades more slowly as the number of incorrectly specified edges increases, with the performance under $\kappa = 0$ being immune to the level of mis-specification.

In summary, as expected, the mis-specification of the prior graph does have an effect on variable selection under the proposed approach, but the degradation in performance (compared to methods which does not include structure knowledge) is not substantial unless the mis-specification level exceeds 25% in some cases. In practice, the proposed approach is best used in scenarios where there is some idea about which connections could be mis-specified in the prior graph, and where the overall mis-specification level is not exceedingly high. One can typically expect the prior knowledge to be correct for a reasonable proportion of edges in genomic applications, for which a high value of tuning parameter can be chosen, whereas for other remaining edges without reliable prior knowledge, it is advisable to choose a low value of the belief parameter or learn it in a data driven manner.

TCGA data analysis: We performed additional data analysis where (a) no graph information was used ($\kappa = 0$); and (b) only 75% confidence was placed on the prior graph knowledge for within platform interactions, which was implemented by setting $(\kappa + a_p)/(\kappa + a_p + b_p) = 0.75$. We present the results in two Tables - Table 2 containing the genes commonly selected in the original analysis (involving a strong belief for within platform interactions, and weak belief for the between platform knowledge) and the genes selected using no prior network knowledge; and Table 3 containing genes commonly selected in the original analysis and the modified analysis corresponding to 75% confidence on the within platform graph. For the first analysis involving no prior network knowledge, 35 genes were selected, whereas for the second analysis, 25 genes were selected. Compared to our original analysis which included 57 important genes, both the additional analysis yielded a smaller number of important genes, potentially suggesting that a weak confidence on the within platform prior knowledge will result in smaller estimated model sizes.

From the Tables 2-3 we see that there is quite a bit of overlap between the three analyses. In particular, we see that 30 out of 35 genes which are selected in the model

with no prior network knowledge are also selected in the original analysis. Out of these 30 genes, 10 are from the copy number variations, 13 are from the mRNA expressions, and the remaining 7 are from methylation. The common genes have similar marginal inclusion probabilities as well as very close effect sizes. In addition, 20 out of the 25 genes selected under the second analysis involving 75% confidence on the within network interactions are common with the original analysis. Out of these common genes, 6 genes are from copy number variation, 10 involve mRNA expressions, and 4 genes involve methylation. Again, the commonly selected genes have similar marginal inclusion probabilities, and the effect are similar as well.

Fig S2. Plots for area under the curve (AUC) for the receiver operating characteristic curve (left) and precision recall characteristic curve (right), under varying belief parameters represented on the x-axis. The prior graph is taken to be the true graph. The solid line with circles represent the AUC for $p = 24$, the dashed line with triangles represent the AUC for $p = 40$, and the dotted line with plus represent the AUC for $p = 80$.

Table 1. Variable selection performance of the proposed approach (BVS-SL) approach under different values of the tuning parameter, in the case where a proportion of edges (denoted by r) are mis-specified. The area under the curve for ROC and PRC curves, denoted as AUC1 and AUC2 respectively, are presented corresponding to varying levels of mis-specification. In particular, we report results when $r = 0, 0.1, 0.25, 0.5, 0.9$ proportion of edges are mis-specified in the prior graph, under Case I(a) and $p = 40$, as described in the simulation section of the manuscript.

**Case I(a) p=40**										
	$r = 0$		$r = 0.1$		$r = 0.25$		$r = 0.5$		$r = 0.9$	
Method	AUC1	AUC2	AUC1	AUC2	AUC1	AUC2	AUC1	AUC2	AUC1	AUC2
BVS-SL(κ=0)	0.974	0.946	0.974	0.946	0.974	0.946	0.974	0.946	0.974	0.946
BVS-SL(κ=5)	0.980	0.980	0.985	0.988	0.982	0.961	0.950	0.961	0.928	0.882
BVS-SL(κ=50)	1.000	1.000	0.969	0.944	0.922	0.867	0.882	0.867	0.867	0.820
Pencred	0.890	0.869	0.890	0.869	0.890	0.869	0.890	0.869	0.890	0.869
SSVS	0.954	0.921	0.954	0.921	0.954	0.921	0.954	0.921	0.954	0.921
Lasso	0.894	0.870	0.894	0.870	0.894	0.870	0.894	0.870	0.894	0.870
EL	0.906	0.874	0.906	0.874	0.906	0.874	0.906	0.874	0.906	0.874
**Case I(b) p=40**										
	$r = 0$		$r = 0.1$		$r = 0.25$		$r = 0.5$		$r = 0.9$	
Method	AUC1	AUC2	AUC1	AUC2	AUC1	AUC2	AUC1	AUC2	AUC1	AUC2
BVS-SL(κ=0)	0.966	0.932	0.966	0.932	0.966	0.932	0.966	0.932	0.966	0.932
BVS-SL(κ=5)	1.000	0.999	0.987	0.975	0.961	0.931	0.900	0.858	0.866	0.818
BVS-SL(κ=50)	1.000	1.000	0.959	0.927	0.904	0.848	0.857	0.805	0.831	0.785
PenCred	0.888	0.790	0.888	0.790	0.888	0.790	0.888	0.790	0.888	0.790
SSVS	0.965	0.897	0.965	0.897	0.965	0.897	0.965	0.897	0.965	0.897
Lasso	0.879	0.770	0.879	0.770	0.879	0.770	0.879	0.770	0.879	0.770
EL	0.890	0.773	0.890	0.773	0.890	0.773	0.890	0.773	0.890	0.773
**Case I(c) p=40**										
	$r = 0$		$r = 0.1$		$r = 0.25$		$r = 0.5$		$r = 0.9$	
Method	AUC1	AUC2	AUC1	AUC2	AUC1	AUC2	AUC1	AUC2	AUC1	AUC2
BVS-SL(κ=0)	0.975	0.948	0.975	0.948	0.975	0.948	0.975	0.948	0.975	0.948
BVS-SL(κ=5)	0.992	0.988	0.981	0.966	0.964	0.937	0.954	0.916	0.941	0.900
BVS-SL(κ=50)	0.995	0.992	0.941	0.911	0.922	0.874	0.975	0.817	0.846	0.791
PenCred	0.749	0.679	0.749	0.679	0.749	0.679	0.749	0.679	0.749	0.679
SSVS	0.842	0.785	0.842	0.785	0.842	0.785	0.842	0.785	0.842	0.785
Lasso	0.630	0.563	0.630	0.563	0.630	0.563	0.630	0.563	0.630	0.563
EL	0.648	0.598	0.648	0.598	0.648	0.598	0.648	0.598	0.648	0.598
0.648	0.598									
**Case I(d) p=40**										
	$r = 0$		$r = 0.1$		$r = 0.25$		$r = 0.5$		$r = 0.9$	
Method	AUC1	AUC2	AUC1	AUC2	AUC1	AUC2	AUC1	AUC2	AUC1	AUC2
BVS-SL(κ=0)	0.959	0.921	0.959	0.921	0.959	0.921	0.959	0.921	0.959	0.921
BVS-SL(κ=5)	0.959	0.956	0.948	0.931	0.929	0.905	0.909	0.879	0.962	0.866
BVS-SL(κ=50)	0.972	0.967	0.928	0.901	0.881	0.835	0.841	0.795	0.809	0.763
PenCred	0.738	0.561	0.738	0.561	0.738	0.561	0.738	0.561	0.738	0.561
SSVS	0.863	0.730	0.863	0.730	0.863	0.730	0.863	0.730	0.863	0.730
Lasso	0.609	0.473	0.609	0.473	0.609	0.473	0.609	0.473	0.609	0.473
EL	0.628	0.536	0.628	0.536	0.628	0.536	0.628	0.536	0.628	0.536

Table 2. Significant genes commonly selected under an analysis specifying a high value of the belief parameter for within platforms and a low value for between platforms, and an analysis with no prior knowledge which is implemented by setting $\kappa = 0$. MIP stands for marginal inclusion probability which quantifies the important of a variable to be selected in the model.

	Original analysis		Analysis with $\kappa = 0$	
Name	MIP	Effect size	MIP	Effect size
HRAS(CN)	0.91	-1.22	0.91	-1.31
AKT2(METH)	0.55	0.04	0.51	0.04
TP53(CN)	0.89	-0.39	0.95	-0.43
CCND1(CN)	0.81	-0.37	0.78	-0.37
CDKN2C(CN)	0.74	0.22	0.65	0.21
IRS1(CN)	0.54	0.13	0.51	0.14
GRB2(METH)	0.73	0.07	0.65	0.06
MDM2(mRNA)	0.71	-0.07	0.65	-0.07
ERBB3(METH)	0.68	-0.07	0.60	-0.06
TP53(METH)	0.68	0.14	0.52	0.11
CDK6(CN)	0.67	0.09	0.50	0.06
IGF1R(mRNA)	0.66	-0.07	0.65	-0.07
TP53(mRNA)	0.65	0.07	0.62	0.07
RAF1(mRNA)	0.63	-0.09	0.66	-0.10
PIK3C2B(CN)	0.53	0.04	0.60	0.05
AKT1(mRNA)	0.63	-0.08	0.58	-0.08
SRC(mRNA)	0.63	0.06	0.55	0.05
FOXO3A(mRNA)	0.53	0.03	0.52	0.03
ERBB2(METH)	0.62	-0.07	0.52	-0.06
PDGFRB(METH)	0.61	-0.06	0.57	-0.07
GRB2(CN)	0.60	0.14	0.60	0.14
NRAS(mRNA)	0.60	0.06	0.60	0.07
PIK3CB(mRNA)	0.51	-0.03	0.62	-0.05
PDGFRB(mRNA)	0.59	0.05	0.51	0.04
MDM2(METH)	0.59	-0.06	0.50	-0.05
SPRY2(CN)	0.58	0.10	0.52	0.09
HRAS(mRNA)	0.58	-0.05	0.62	-0.06
ERBB2(mRNA)	0.58	0.04	0.51	0.03
PTEN(mRNA)	0.50	0.03	0.51	0.04
EGFR(CN)	0.55	0.07	0.56	0.06

Table 3. Significant genes commonly selected under an analysis specifying a high value of the belief parameter for within platforms and a low value for between platforms, and an analysis specifying 75% prior confidence on within platform interactions and low confidence on between platform interactions. MIP stands for marginal inclusion probability which quantifies the important of a variable to be selected in the model.

	Original analysis		Analysis with 75% confidence	
Name	MIP	Effect size	MIP	Effect size
HRAS(CN)	0.91	-1.22	0.90	-1.35
TP53(CN)	0.89	-0.39	0.94	-0.44
CCND1(CN)	0.81	-0.37	0.75	-0.36
CDKN2C(CN)	0.74	0.22	0.63	0.22
GRB2(METH)	0.73	0.07	0.63	0.07
MDM2(mRNA)	0.71	-0.07	0.66	-0.07
ERBB3(METH)	0.68	-0.07	0.58	-0.06
TP53(METH)	0.68	0.14	0.52	0.11
IGF1R(mRNA)	0.66	-0.07	0.60	-0.07
TP53(mRNA)	0.65	0.07	0.56	0.06
RAF1(mRNA)	0.63	-0.09	0.64	-0.10
AKT1(mRNA)	0.63	-0.08	0.52	-0.06
SRC(mRNA)	0.63	0.06	0.52	0.05
FOXO3A(mRNA)	0.53	0.03	0.50	0.03
PDGFRB(METH)	0.61	-0.06	0.56	-0.06
GRB2(CN)	0.60	0.14	0.57	0.14
NRAS(mRNA)	0.60	0.06	0.59	0.07
PIK3CB(mRNA)	0.51	-0.03	0.60	-0.05
HRAS(mRNA)	0.58	-0.05	0.63	-0.06
EGFR(CN)	0.55	0.07	0.54	0.07
